# Optimized bisulfite sequencing analysis reveals the lack of 5-methylcytosine in mammalian mitochondrial DNA

**DOI:** 10.1186/s12864-023-09541-9

**Published:** 2023-08-04

**Authors:** Zhenyu Shao, Yang Han, Dan Zhou

**Affiliations:** 1 State Key Laboratory of Molecular Biology, Shanghai Institute of Biochemistry and Cell Biology, Center for Excellence in Molecular Cell Science, Chinese Academy of Sciences, University of Chinese Academy of Sciences, Shanghai, 200031 China; 2 Shanghai Key Laboratory of Medical Epigenetics, Institutes of Biomedical Sciences, Shanghai Medical College of Fudan University & Chinese Academy of Medical Sciences (RU069), Shanghai, 200032 China; 3 Center for Medical Research and Innovation, Shanghai Pudong Hospital, Shanghai Key Laboratory of Medical Epigenetics, Institutes of Biomedical Sciences, Shanghai Medical College of Fudan University & Chinese Academy of Medical Sciences (RU069), Shanghai, 201399 China

**Keywords:** Mitochondria, DNA methylation, NUMTs, Bisulfite sequencing

## Abstract

**Background:**

DNA methylation is one of the best characterized epigenetic modifications in the mammalian nuclear genome and is known to play a significant role in various biological processes. Nonetheless, the presence of 5-methylcytosine (5mC) in mitochondrial DNA remains controversial, as data ranging from the lack of 5mC to very extensive 5mC have been reported.

**Results:**

By conducting comprehensive bioinformatic analyses of both published and our own data, we reveal that previous observations of extensive and strand-biased mtDNA-5mC are likely artifacts due to a combination of factors including inefficient bisulfite conversion, extremely low sequencing reads in the L strand, and interference from nuclear mitochondrial DNA sequences (NUMTs). To reduce false positive mtDNA-5mC signals, we establish an optimized procedure for library preparation and data analysis of bisulfite sequencing. Leveraging our modified workflow, we demonstrate an even distribution of 5mC signals across the mtDNA and an average methylation level ranging from 0.19% to 0.67% in both cell lines and primary cells, which is indistinguishable from the background noise.

**Conclusions:**

We have developed a framework for analyzing mtDNA-5mC through bisulfite sequencing, which enables us to present multiple lines of evidence for the lack of extensive 5mC in mammalian mtDNA. We assert that the data available to date do not support the reported presence of mtDNA-5mC.

**Supplementary Information:**

The online version contains supplementary material available at 10.1186/s12864-023-09541-9.

## Background

The most prevalent DNA modification in the mammalian nuclear genome is 5-methylcytosine (5mC), which is mainly found in symmetric CpG dinucleotides and represents approximately 1 to 4% of total cytosines [1–3]. Nuclear 5mC plays a critical role in a variety of biological processes, including the regulation of gene expression, genomic imprinting, X inactivation, and the suppression of repetitive elements [4, 5]. The presence of nuclear 5mC in most eukaryotes has led to a search of its existence in mitochondrial DNA (mtDNA). Although the nuclear DNA methylation has been extensively characterized, the prevalence and function of 5mC in mtDNA remains controversial to date.

The human mtDNA is a ~16.5 kb double-stranded, circular molecule, presenting in multiple copies per mitochondrion [6]. The mitochondrial genome encodes 13 proteins involved in respiratory chain complexes as well as 2 ribosomal RNAs and 22 transfer RNAs specific to this organelle [7]. All other 1000–3000 mitochondrial proteins, including those required for mtDNA replication and transcription, are encoded by the nuclear genome and imported into the mitochondria using specific mitochondrial targeting sequence (MTS) [8]. In contrast to the nuclear DNA that associates with histones, mtDNA is packaged into individual nucleoids consisting of DNA-binding proteins and peripheral factors, which facilitate mtDNA replication and gene expression [9–11]. However, while the existence of 5mC in mtDNA remains uncertain, the dysregulation of 5mC has been widely associated with the development of cancer, neurodegenerative diseases, and various physiological or pathological conditions [12–16].

A range of experimental approaches have been applied to detect and quantify 5mC in mammalian mtDNA. Using radiolabeling, it was first reported in the 1970s that about 0.2 ~ 0.6% of cytosines are methylated in mtDNA from various mammalian cells [17]. Since then, methods based on radiolabeling, 5mC-sensitive restriction enzymes, 5mC-specific antibodies, and mass spectrometry have been employed to evaluate the level of 5mC in mammalian mtDNA. Two studies using 5mC-sensitive restriction enzymes, MspI and HpaII, revealed a highly restricted distribution of 5mC exclusively within the CpG dinucleotide context, occurring at a frequency of 2 to 5% (equivalent to less than 1% of 5mC/C) in mtDNA from mouse and human cells [18, 19]. Leveraging liquid chromatography-tandem mass spectrometry (LC-MS/MS), it was shown that the level of 5mC in mtDNA increased markedly, from 13 to 25%, in lymphoblastoid cells obtained from individuals with Down syndrome compared to control cells [20]. More recently, sequencing-based methods enabled the detection of 5mC at genome-scale. With methylated DNA immunoprecipitation-sequencing (MeDIP-Seq), an antibody-based method, a conserved pattern of 5mC was observed near gene start sites across various cell and tissue types in mtDNA [21].

Another commonly used approach to quantify 5mC at single-base resolution is bisulfite sequencing [16, 22–29]. By DNA pyrosequencing, a study reported 2 to 18% 5mC in the D-loop and 16S rRNA gene in mtDNA isolated from mouse brain, liver, and testis [23]. Lately, 5mC in mtDNA was mapped at single-base resolution and in a strand-specific manner using whole genome bisulfite sequencing (WGBS) [16, 24]. Comprehensive bioinformatic analyses revealed extensive methylation of human mitochondrial genomes and a distinctive pattern of 5mC in normal and cancer cells [16]. In another study, 5mC was shown to be strongly biased toward the light (L)-strand and the non-CpG motif, with conserved peak loci [24]. The quantification of mtDNA-5mC was generally found to be more than 20% and was shown to be modulated by DNA methyltransferases (DNMTs), the canonical nuclear DNA methyltransferase, and was inversely associated with mitochondrial transcription [16, 24]. Collectively, these studies have reported the presence of a wide range of 5mC levels (ranging from 0.2 to 25% 5mC/C) in mammalian mtDNA [30].

Although a number of studies have reported the presence of 5mC in mammals mtDNA, several other research groups were unable to detect 5mC or confirm its existence in mammalian mtDNA with various methods, thus casting doubt on the existence and levels of mtDNA-5mC. Using radiolabeling and two-dimensional thin-layer chromatograph, 5mC in HeLa mtDNA was estimated to be less than 0.05 mol percent of total mitochondrial nucleotides, while that in nuclear DNA was 0.7 [31]. In another study, the levels of mtDNA-5mC from mouse liver were estimated to be between 0.3% and 0.5% by mass spectrometry, but this estimation was considered to be confounded by contaminations with nuclear DNA, whose 5mC content is about 6% of cytosines [32]. Using both region-specific and genome-wide approaches in HEK293 and HCT116 cell lines, a group concluded that CpG methylation is absent in mtDNA [33].

The conflicting views on mtDNA methylation can be partly attributed to technical artifacts and limitations associated with studying this modification [34]. It has been shown that mtDNA fragments that are less engaged in the mtDNA supercoiled structure are preferably freed during sonication and ligated to sequencing adapters, resulting in an overrepresentation of these fragments. Additionally, the secondary structure of mtDNA may hinder the accessibility of sodium bisulfites, leading to poor conversion and an overestimation of methylation levels [35]. Cytosine content is another leading cause of sequencing bias. Bisulfite reactions have been known to induce template degradation by triggering depyrimidination of bisulfite adducts formed through sulfonation and deamination of cytosine [36]. This leads to a depletion of cytosine-rich DNA and unmethylated fragments from the total library, resulting in a skewed representation of genomic sequences and an overestimation of 5mC levels [37]. Indeed, the majority of bisulfite library preparation methods have been found to yield significantly biased sequence outputs, leading to an overestimation of DNA methylation [37]. In addition to bisulfite-related false positives, mapping to highly similar nuclear mitochondrial DNA sequences (NUMTs) also introduces misalignment artifacts [38, 39], which can lead to artifactual signals of 5mC.

Recently, mtDNA-5mC was reinvestigated with bisulfite-independent single-molecule Oxford Nanopore Sequencing (ONS) [40–43]. However, these studies have not yet reached a consensus. While the presence of mtDNA methylation was reported in cultured cancer cell lines [40], human blood cells [41], and human primary normal and tumor tissues [42], another study suggested that the mtDNA-CpG methylation signals detected by ONS could be attributed either to sequence-specific false positives introduced by the technique or were below the error threshold modelled using negative controls [43].

In this study, we aimed to address this controversial question by performing comprehensive bioinformatic analyses on both published and in-house WGBS datasets. Our analysis showed that strand-biased sequencing reads gave rise to false positive 5mC signals in mtDNA, while reads of NUMTs interfered with the quantification of mtDNA-5mC. We therefore proposed an optimized procedure for library preparation and data analysis of mtDNA methylation with bisulfite, in which mtDNA should be linearized to ensure efficient bisulfite conversion for Sanger bisulfite sequencing and sequencing reads should be examined for depth and NUMTs for WGBS. With this modified procedure, we identified the sources of false positive mtDNA-5mC signals. Combining our findings with TET-assisted pyridine borane sequencing (TAPS) and Enzymatic Methyl-seq (EM-seq) data, predictions of MTS, and analysis of the mitochondrial proteome, we provide new evidence for the lack of 5mC in mammalian mtDNA.

## Results

### Severe strand-specific sequencing biases introduce artifactual signals of mtDNA 5mC

**Fig. 1:**
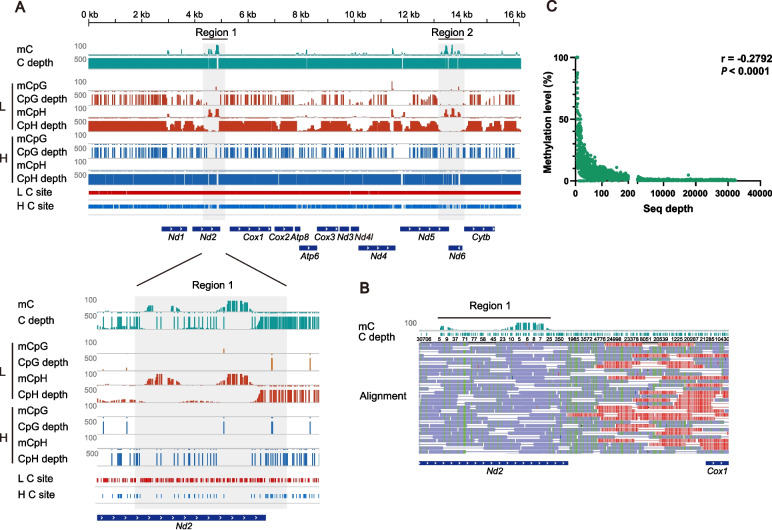
Strand-specific sequencing bias causes artifactual mtDNA-5mC signals. **A** Re-analysis of a published WGBS dataset (GSE56697) demonstrates L strand-specific 5mC signals in the mtDNA of mouse oocyte. Green tracks show methylation level (top, scale from 0 to 100) and sequencing depth for each cytosine (bottom, scale from 0 to 500) in the double-stranded mtDNA. Methylation levels and sequencing depths of CpG and CpH were analyzed separately for L (red tracks) and H (blue tracks) strand. Cytosine sites in each strand were marked on the bottom. Region 1 and Region 2 harbor high 5mC signals. A close-up view of Region 1 is shown to the bottom, in which the 5mC signals reflect methylation of CpH sites with extremely low sequencing depth in the L strand. Coverage ≥ 3 for 5mC, and ≥ 1 for mCpG and mCpH on each strand. **B** An IGV snapshot of the sequencing reads showing that Region 1 is devoid of reads aligned to the L strand. Reads aligned to the L strand are colored in red and to the H strand in blue. C to T and G to A signals are marked by red and green vertical lines respectively. **C** Sequencing depth and methylation ratio (5mC/C %) in the oocyte are inversely correlated (*P* < 0.001). Each dot refers to one cytosine site in the mtDNA

A previous study showed that mtDNA-5mC signals were strongly biased to the L strand and non-CpG context across different cell types and developmental stages using published WGBS datasets [24]. To validate their findings, we re-analyzed mtDNA methylation levels using Bismark mapping tools for the same datasets, which included mouse primordial germ cells (PGCs), oocyte, sperm, early embryos at different developmental stages (GSE56697) [44], and prefrontal cortex (PFC) (GSM830249) [45]. 5mC signals were observed in the mtDNA of oocyte, E6.5 embryo, and sperm (Fig. S1A-B). We examined the sequencing reads aligned to the mitochondrial genome in a strand-specific manner and observed a predilection of 5mC signals at CpH sites on the L strand, localized specifically at gene boundaries (Fig. 1A for oocyte, Fig. S1C for E6.5). These findings are in agreement with a previous report [24]. However, our further analysis revealed that the sequencing depth of cytosines in Region 1 and 2 (< 10) was far lower compared to both the average depth (10,221) and the depth observed in adjacent regions (Fig. 1A, B). In addition, a severe inverse correlation was observed between the sequencing depth and the mtDNA methylation level, especially when the depth fell below 100 (Fig. 1C). A lower depth is expected for the cytosine-rich L strand (3976 cytosines) than for the H strand (2013 cytosines) since bisulfite-induced fragmentation usually occur at unmethylated cytidines [36]. A previous study that compared multiple WGBS libraries revealed an L/H ratio ranging from 10 to 40% [37]. Consistent with this, we observed that the mean depth of the L strand was considerably lower than that of the H strand in oocyte in this dataset (2752 versus 24141, Table S1).

To exclude the possibility that 5mC signals and biased sequencing depth were due to bisulfite alignment tools, we analyzed these data with BSseeker2 independently. Comparing the mapping and calling results of BSseeker2 to those of Bismark revealed a highly similar methylation profile, with strong 5mC signals presenting in oocytes and E6.5 embryos (Fig. S2A-D) particularly in regions with low sequencing depth (Fig. S2C). Again, inverse correlations were observed between depth and methylation level using BSseeker2 (Fig. S2E).

These biased sequence representations, both between strands and within strands, are likely due to two factors 1) bisulfite-induced DNA degradation of cytosine-rich genomic regions [37], and/or 2) overrepresentation of mtDNA fragments that do not undergo supercoiling [35, 46]. There is a substantial difference in cytosine content between the L and H strands of mtDNA. Bisulfite treatment induces degradation of DNA that is enriched for unmethylated cytosines, resulting in regions with fewer cytosines or those exposed less to bisulfite being over-represented in the sequencing library [36, 37]. In Region 1, while cytosine is not highly enriched in the L strand, it is nearly depleted from the H strand. This has led to an underrepresentation of reads from the L strand (123) compared to the H strand (439497). In addition, regions involved in secondary structures tend to be underrepresented in the library because they are less freed during sonication and are also less accessible to bisulfite. In these low coverage regions, even a small number of poorly converted reads can have a significant impact on the measurement of 5mC. Consequently, the reported 5mC signals are not reliable evidence to support the existence of 5mC in mouse mitochondrial DNA.

Because methylation measurements at specific genomic regions can vary substantially for the same cell type due to experimental procedures, we wondered whether the same problem occurs with mtDNA methylation. We noticed a published WGBS data series in which the methylation levels of mtDNA from four HEK293T biological replicates [47] were very dissimilar (ranging from 1.14 to 10.16%) even they underwent the same processing procedures (Fig. S3A-C). While three replicates showed very low levels of mtDNA-5mC, only the replicate GSM2425586 displayed high mtDNA-5mC signals throughout the mtDNA. This replicate also exhibited a severe strand depth bias (H/L = 26.59) (Table S1).

Together, our analyses indicate that the reported mtDNA-5mC is a classic case of artefact resulting from a severe strand-specific sequencing bias.

### Linearization of mtDNA eliminates false positive 5mC signals

**Fig. 2:**
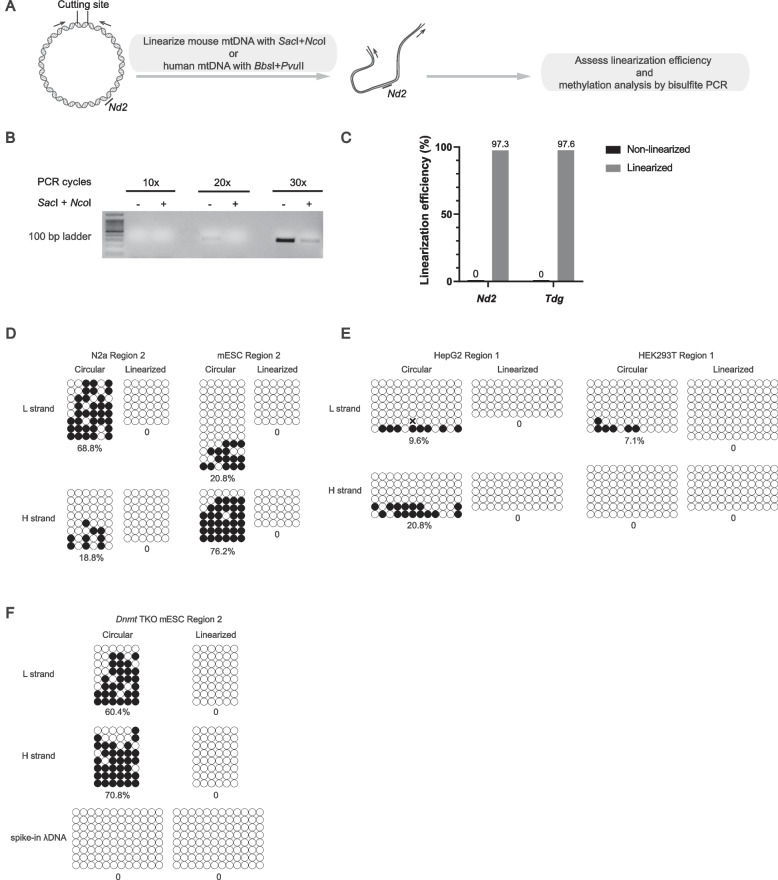
Complete linearization of mtDNA eliminates false-positive 5mC signals. **A** Schematic showing the workflow of linearization and methylation analysis of mtDNA. Two restriction enzymes with adjacent target sites are used to linearize human and mouse mtDNA prior to bisulfite treatment. Arrows, PCR primers flanking the cutting sites. **B** PCR analysis of the linearization efficiency of mtDNA from N2a cells using primers flanking the cutting sites. The original image of uncropped DNA gel was shown in Fig. S8. **C** Confirmation of linearization efficiency of N2a mtDNA by qPCR analysis. Primers flanking the cutting sites are designed to amplify the 242 bp region targeted by the restriction enzymes. Mitochondrial gene *Nd2* and nuclear gene *Tdg* are chosen as internal control to independently calculate the linearization efficiency. **D**-**F** Methylation analysis of Region 2 in the mtDNA of N2a and mouse embryonic stem cell lines (mESC) (**D**), Region 1 in HEK293T and HepG2 cell lines (**E**) and Region 2 in *Dnmt* triple knockout (TKO) mESC cell lines (**F**) by site-specific bisulfite sequencing with or without linearization. Results of L and H strands are shown separately. Complete bisulfite conversion of spike-in λDNA with or without linearization suggests that the conversion efficiency of linear spike-ins does not reflect that of the circular mtDNA

The mtDNA genome is organized in coiled and supercoiled secondary structures [46]. Previous studies have demonstrated that removing supercoiled structures from mtDNA using the restriction enzyme *Bam*HI can significantly increase the bisulfite conversion rate and, subsequently reduce the presence of artifactual 5mC signals [35, 48]. To independently determine the impact of circular structure on methylation analysis of mtDNA, intracellular DNA was extracted using phenol-chloroform from a mouse neuroblastoma cell line Neuro-2a (N2a) and then digested with two restriction endonucleases, *Sac*I and *Nco*I. These endonucleases target adjacent sites in the mouse mtDNA (Fig. 2A). Primers flanking the targeting sites were used to assess the linearization efficiency. Agarose gel analysis (Fig. 2B) and qPCR (Fig. 2C) both confirmed that the linearization of mtDNA was highly efficient (no less than 97.3%). Next, we examined methylation levels at Region 2 in a strand-specific manner using linearized mtDNA from N2a and mouse embryonic stem cells (mESC). For both the L and H strands, the 5mC signals were no longer detectable after linearization (Fig. 2D). We further tested the methylation levels of mtDNA derived from HepG2 hepatocarcinoma cells, which were reported to have more than 40% methylation in the mitochondria genome [16]. However, we observed much fewer 5mC signals in the circular mtDNA and no methylation signals after linearization (Fig. 2E left). A similar trend was observed for mtDNA of human embryonic kidney cells HEK293T (Fig. 2E right). The presence of false positive 5mC signals in the circular but not linearized mtDNA of *Dnmt* triple knockout (TKO) mESC (Fig. 2F), in which both mitochondrial and nuclear genome should be devoid of 5mC, further supports that linearization prior to bisulfite conversion is essential to detect true mtDNA-5mC signals. Importantly, the bisulfite conversion rate of the spike-in λDNA was 100% for both circular and linearized forms, suggesting that the conversion efficiency of linear spike-ins does not reflect that of circular mtDNA (Fig. 2F, bottom). In sum, our results suggest that linearization of mtDNA improves the efficiency of bisulfite conversion and completely eliminates false positive 5mC signals.

### MtDNA-5mC is below detection limit or even absent in high throughput sequencing data

**Fig. 3:**
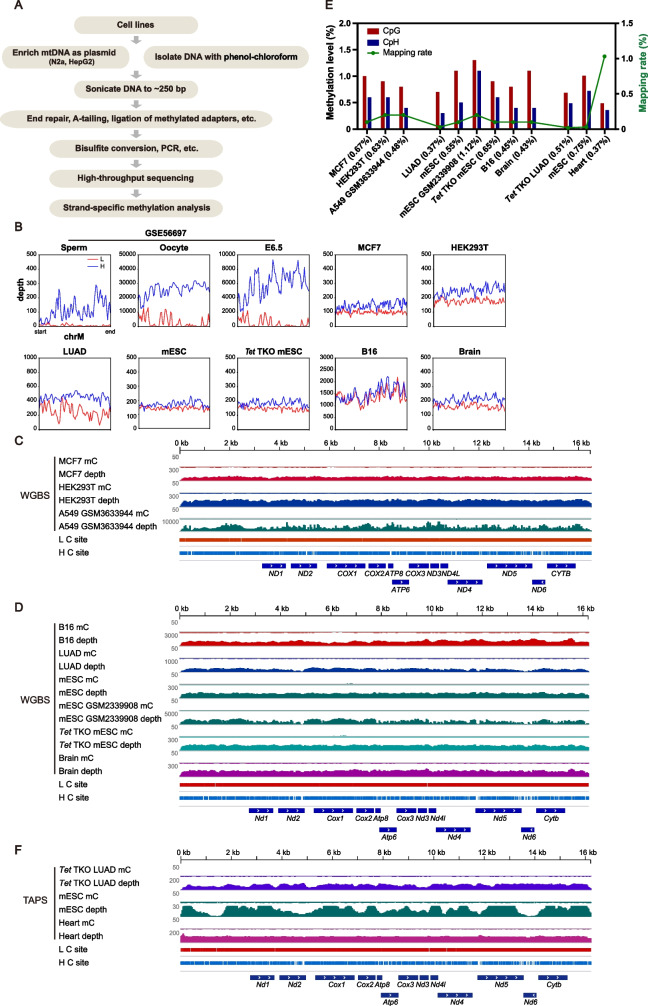
High-throughput sequencing reveals the lack of 5mC in mammalian mtDNA. **A** Schematic showing the workflow of mtDNA enrichment, fragmentation, and whole genome bisulfite sequencing (WGBS). **B** Metaplots for the sequencing depths across the mtDNA of published (mouse oocyte, sperm and E6.5 embryo from GSE56697) and our own WGBS data. **C**, **D** Methylation levels and sequencing depths of mtDNA in selected human (**C**) and mouse (**D**) cell lines measured by WGBS. 5mC signals are extremely low across the entire mtDNA. **E** Bar graph showing CpG and CpH methylation levels (left y-axis) and mapping rates (green line, right y-axis) for indicated cell and tissue types. Average methylation levels are shown in the parentheses. **F** Methylation levels and sequencing depths of mtDNA in selected mouse cell lines and tissues measured by TAPS. 5mC signals are extremely low across the entire mtDNA

Because cytosines in the CpH context may also be methylated in mtDNA, quantification of 5mC with classic Sanger bisulfite sequencing could be misleading at times due to the amplification bias introduced by non-degenerate primers. To avoid such confusion, we performed WGBS to map mtDNA-5mC at single-base resolution in several human and mouse cell lines. Methylation of mtDNA has been detected in HepG2 and MCF7 cells [16], but not in HEK293T cells [33]. We therefore independently prepared WGBS libraries and examined mtDNA-5mC levels in these cell lines, as well as in several mouse cell lines including N2a, B16, a lung adenocarcinoma cell line (LUAD), mESC, *Tet* TKO mESC, and mouse brain tissue. The mtDNA was isolated using the phenol-chloroform method, and for N2a and HepG2 cells, mtDNA was also enriched as plasmids [49] (Fig. 3A). Following extraction, the mtDNA was fully fragmented by sonication.

The sequencing reads of these in-house WGBS data were evenly distributed throughout the mtDNA (Fig. 3B-D), and the depth ratios (H/L) were less than 2 (Table S1). We did not observe any strong 5mC signals across the entire mitochondrial genome in these cells (Fig. 3C, D), which disagrees with the previous report of mtDNA methylation in HepG2 and MCF7 cell lines [16]. We showed that both the CpG and CpH contexts contained no more than 1.1% 5mC, with the average methylation levels ranging from 0.37 and 0.67% in our own WGBS data (Fig. 3E). Since the bisulfite conversion rates usually do not exceed 99%, the 5mC signals below 0.67% are likely the result of incomplete bisulfite conversion. Moreover, we analyzed human platelets isolated from cord blood using WGBS. Platelets are cytoplasmic pieces formed from megakaryocytes and therefore, do not contain nuclear materials [50]. Our analysis showed that the average methylation level was no more than 0.59% (Fig. S4A), and the reads were evenly distributed (Fig. S4B-C, Table S1).

Having failed to detect reliable mtDNA-5mC in cell lines and primary cells, we sought to verify our WGBS results by TAPS [51], a bisulfite-free methylation mapping approach, using *Tet* TKO LUAD cells, mESC, and mouse heart tissues. Consistent with our previous results, the average methylation ratio ranged from 0.37% to 0.75% in the mtDNA from these cells (Fig. 3E, F), and there was no observed bias in strand depth (data not shown). Because the false positive rate of TAPS (0.23 ~ 1.63%) is comparable to that of WGBS (~1%) [51], we cannot distinguish between real 5mC signals and background noise. Furthermore, since 5mC does not accumulate in mtDNA from *Tet* TKO mESC and *Tet* TKO LUAD cells (Fig. 3E), it seems that TETs are not involved in the epigenetic regulation of mtDNA.

We also analyzed mtDNA methylation using published EM-seq data with NA12878 DNA [52]. EM-seq purely uses enzymes to detect 5mC and its oxidized forms. Like TAPS, EM-seq bypasses bisulfite treatment-induced DNA loss and sequencing bias introduced by bisulfite treatment [52]. Our EM-seq analysis showed that there were no signals for 5mC in mtDNA, which is consistent with the bisulfite sequencing results of NA12878 DNA (Fig. S5A). Compared to bisulfite libraries, EM-seq libraries showed a more even distribution within the strands and between the H and L strands of mtDNA (Fig. S5A-B).

Collectively, our data indicate that the methylation levels of mtDNA in mammalian cells, if ever present, are far lower than previously reported [16, 24], and are below the detection limit (< 0.75%).

### Interfering signals from NUMTs lead to erroneous detection of mtDNA-5mC

**Fig. 4:**
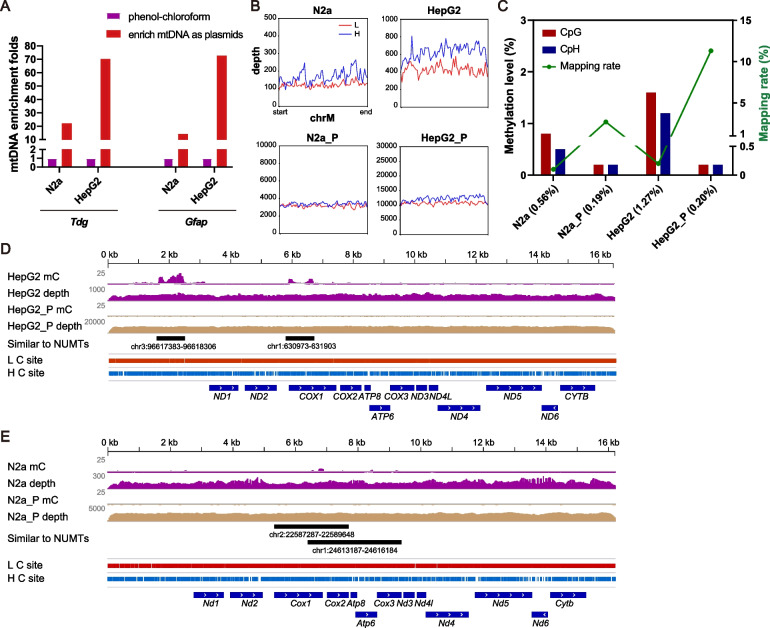
Enrichment of mitochondrial DNA improves the mapping rate and reliability of WGBS data. **A** qPCR analysis of genomic DNA was used to examine the enrichment efficiency of mtDNA over the nuclear DNA from N2a and HepG2 cell lines using mitochondrial and nuclear specific primers. Nuclear genes *Tdg* and *Gfap* are independently used as internal control. **B** Metaplots for the sequencing depths across the mtDNA of N2a and HepG2 cell lines. **C** Bar graph showing CpG and CpH methylation levels (left y-axis) and mapping rates (green line, right y-axis) for N2a and HepG2 cell lines. Average methylation levels are shown in the parentheses. **D**, **E** Methylation levels and sequencing depths of mtDNA in HepG2 (**D**) and N2a (**E**) cell lines measured by WGBS

The phenol-chloroform method usually yields a low amount of mtDNA and is often contaminated with a large amount of nuclear DNA. To analyze mitochondrial DNA methylation, it is reasonable to employ an enrichment step to obtain mitochondrial-focused sequencing data while also avoiding interferences from nuclear sequences. Genomic DNA-qPCR analysis showed that the enrichment of mtDNA over nuclear DNA by the plasmid extraction method was 15 to 70 times higher than by the phenol-chloroform method in N2a and HepG2 cells (Fig. 4A). Notably, when analyzing the WGBS data for N2a and HepG2, we found that enriching mtDNA in HepG2 and N2a cells using the plasmid extraction method (hereafter HepG2_P and N2a_P) produced a less biased distribution of reads (Fig. 4B, Table S1), higher mapping rates, and lower 5mC levels (Fig. 4C, from 0.56% to 0.19% in N2a and 1.27% to 0.20% in HepG2).

Next, we examined the 5mC signals in the IGV genome browser. We found that these signals were localized in NUMTs-homologous mtDNA sequences. When mtDNA was enriched, these signals disappeared (Fig. 4D-E). NUMTs are known to be positive for 5mC signals [53], and the misalignment of nuclear sequences to mtDNA has been suggested to influence the quantification mtDNA methylation [35, 43, 54]. In agreement with this notion, our results suggest that contamination from NUMTs may contribute to the false 5mC signals observed in mtDNA. Enrichment of mtDNA prior to high-throughput sequencing effectively reduces these false positive signals and provides a high number of usable reads, thereby increasing the coverage of the mitochondrial genome.

Mitochondrial and NUMTs sequences are homologues due to their common origin. For high-throughput sequencing analysis of mtDNA, including only reads that uniquely mapped to mtDNA [24] or setting the Phred-scaled minimal mapping quality at 30 (equivalent to a 99.9% alignment accuracy) [55] were used to prevent the interfering signals from NUMTs. However, these methods are more effective for human than for mouse, where certain mitochondrial sequences are identical to NUMTs. For example, the sequence of mouse chrM:6394-9458 (*Cox1* to *Cox3*) is exactly the same as that of chr1:24613120-24616184, making the reads indistinguishable in next generation sequencing.

**Fig. 5:**
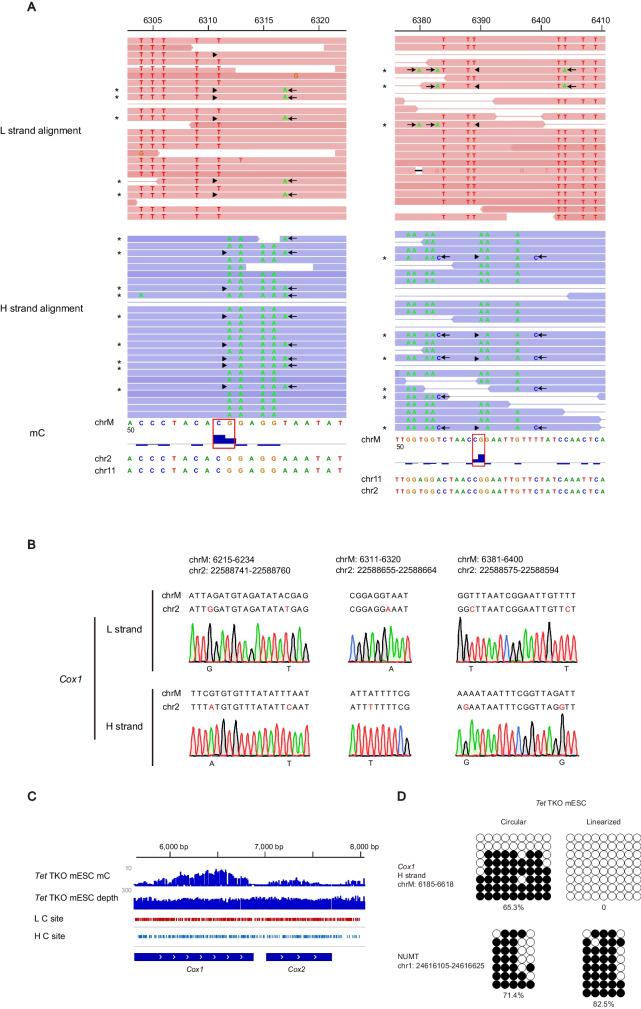
5mC signals at *Cox1* gene locus in the mtDNA of mouse sperm and *Tet* TKO mESC come from NUMTs. **A** Two IGV snapshots showing the WGBS reads (GSE56697, mouse sperm) aligned to *Cox1*. Reads aligned to the L strand are colored in red, to the H strand in blue. In the aligned reads, bases unmatched with mitochondrial genome are shown in colored letters and are divided into two categories as follows, one comes from the bisulfite conversion of unmethylated Cs, the other is single base differences (SBDs) naturally exist between mtDNA and NUMTs (arrow). The unconverted Cs (arrowhead) and SBDs usually lie within the same read (marked with asterisks), suggesting the 5mC signals in mtDNA (red line box) are false positive calls due to misalignment. Potential alternative genomic sequences for NUMTs are shown underneath. For the left panel, misaligned reads are identical to NUMTs on chr2 and chr11. For the right panel, reads misaligned to the L strand are identical to NUMTs on chr11, to the H strand identical to NUMTs on chr2. **B** Sanger bisulfite sequencing towards *Cox1* in mouse sperm. L and H strands are shown separately. Non-SBD-Cs in the non-CpG context of the reference sequences are masked as Ts. SBDs between mtDNA and NUMTs are shown in red font. In the case of C/T SBDs which were both read as Ts in bisulfite sequencing, the sequence of adjacent regions or the opposite strand helps to distinguish mtDNA vs. NUMTs origin of reads. The BS-PCR products align strictly to one of the NUMTs in chr2 instead of to *Cox1* in mtDNA. **C** An IGV snapshot showing 5mC signals at *Cox1* gene locus in the *Tet* TKO mESC. **D** Methylation analysis of *Cox1* from *Tet* TKO mESC by Sanger bisulfite sequencing with or without linearization of the mtDNA. Primers were designed to distinguish *Cox1* and NUMTs

Normally, there are between 1000 and 10 000 copies of mtDNA in each mammalian cell [56], hence the probability of amplifying similar NUMTs is extremely low due to the vast excess of mtDNA. However, once methylated, these nuclear sequences can be over-represented after bisulfite-induced degradation of unmethylated mtDNA sequences and during PCR of converted DNA fragments [37]. This becomes even more problematic in cells containing fewer copies of mtDNA, such as sperm (10–100) [57, 58]. In the published WGBS dataset (GSE56697), 5mC signals were observed in the CpG context of mouse sperm mtDNA at *Cox1* and *Cox2* gene loci (Figs. S1A-B and S2A-B). Since the sequence of this region is identical to that of NUMTs, we directed our attention to the 5’ upstream sequence that contains single-base-differences (SBDs) between mtDNA and NUMTs. Upon examining each single read mapped to this region, we found that unconverted cytosines, which gave rise to 5mC signals, always appeared in the reads with SBDs. This observation suggests that the methylation calls were erroneous due to misalignment (Fig. 5A). Through sequence alignment (BLAT function in IGV), we found that the sequences of these misaligned reads were identical to those of NUMTs from chr2: 22588652-22588672 and chr11: 90539030-90539050 (Fig. 5A, left panel), or chr11: 90538942-90538977 and chr2: 22588564-22588599, respectively (Fig. 5A, right panel). These observations suggest that the 5mC signals most likely reflect the methylation status of NUMTs rather than that of mtDNA. In high-throughput sequencing analysis, reads mapping usually allow a certain level of mismatch, which would inevitably introduce erroneous 5mC signals due to NUMTs. This could potentially explain the methylation signals observed across the *Cox1* and *Cox2* gene loci, as well as other mtDNA regions.

To verify the methylation levels at these regions in mouse sperm mtDNA, we applied Sanger bisulfite sequencing towards *Cox1*. The bisulfite PCR products share an identical sequence to the NUMTs found on mouse chromosome 2 (Fig. 5B), suggesting that sperm mtDNA, in comparison to nuclear DNA, was at a disadvantage during PCR amplification. Similarly, the amplicons of regular PCR were mainly derived from NUMTs (Fig. S6). Thus, the presence of NUMTs can lead to false quantification of methylation levels, especially in cells with a limited number of mtDNA copies.

We noticed subtle 5mC signals (chrM: 6100-6800, ~5 to 10%) across the *Cox1* gene locus in *Tet* TKO mESC (Fig. 5C). To distinguish between *Cox1* and NUMTs, we designed primers for Sanger bisulfite sequencing. We found that the 5mC signals, which cannot be eliminated by linearization of mtDNA, actually originated from NUMTs on mouse chromosome 1 (Fig. 5D). This indicates that NUMTs can contribute to false mtDNA-5mC signals, independent of bisulfite conversion efficiency and sequencing bias. Removing the nucleus is expected to eliminate contamination from NUMTs during methylation analysis of mitochondrial DNA. We performed a micromanipulation to remove nuclear and polar body DNA from mouse oocytes (Fig. S7A) and then examined *Nd6* gene locus using Sanger bisulfite sequencing. As expected, we found that the 5mC signals were no longer detectable (Fig. S7B), confirming that the 5mC signals observed in this region were false positives (Fig. S7C).

Collectively, our results suggest that the observed 5mC signals in mouse sperm and *Tet* TKO mESC are erroneous detection caused by interfering signals from NUMTs. While enrichment of mtDNA before sequencing is expected to reduce such false signals, total DNA isolated with phenol-chloroform can also provide reliable detection of mitochondrial DNA methylation by WGBS, as long as the sequencing depth is not severely biased and the interference from NUMTs signals is carefully examined.

### 5mC-associated proteins and co-factors are absent in mitochondria

**Fig. 6:**
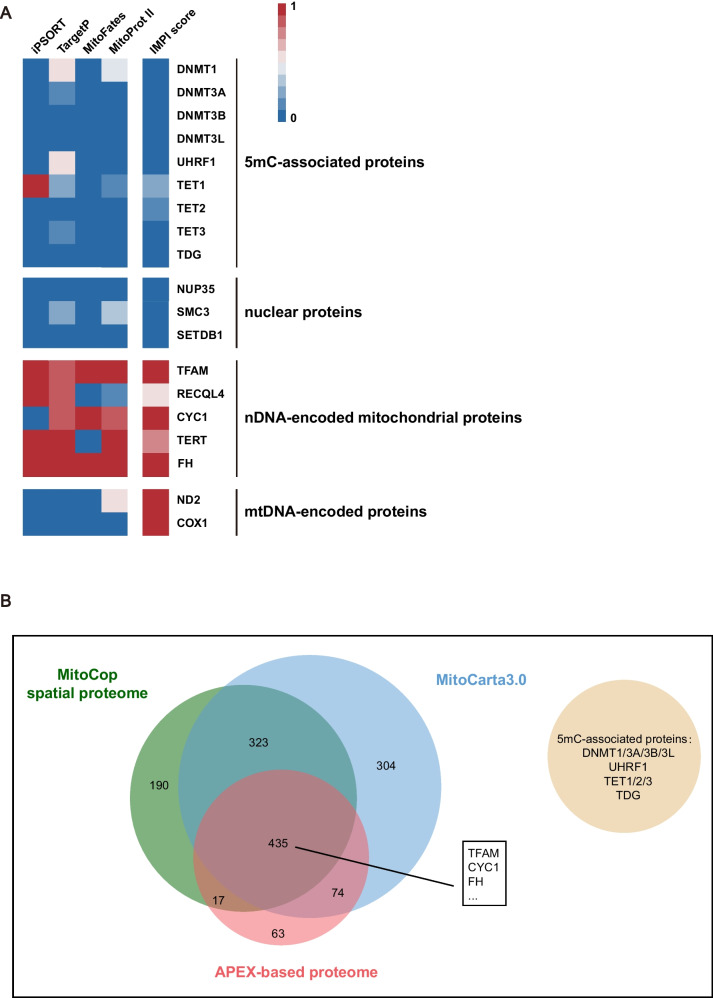
5mC-associated protein and co-factors are absent in mitochondria. **A** A heatmap showing the MTS scores using different prediction tools. Nuclear proteins are used as negative controls while nuclear DNA-encoded mitochondrial proteins as positive controls. On a scale of 0 to 1, a higher score indicates that the protein is more likely to locate in the mitochondrial (IMPI) or contain at least one functional mitochondrial targeting sequence (MTS) (iPSORT, TargetP, MitoFates and MitoProt). **B** A Venn diagram showing that 5mC-associated proteins are not among the mitochondrial proteins identified by three independent mitochondrial proteome databases. Known mitochondrial proteins, such as TFAM, CYC1 and FH, are consistently identified in all three databases

Recent studies have reported the presence of DNA modification proteins in mammalian mitochondria [16, 23, 24, 29, 59–61]. For example, the mitochondrial targeting sequence (MTS) of mouse and human DNMT1 was predicted by MitoProt II, and was detected by immunoblots and immunofluorescent assays in 2011 [59]. However, several other groups showed that DNMT3A, rather than DNMT1, is the protein responsible for regulating mtDNA methylation within the mitochondria [23, 24, 60]. Because we did not observe any reliable 5mC signals in the mtDNA, we sought to re-inspect human 5mC-associated proteins using a variety of MTS prediction tools, namely iPSORT [62], target [63], MitoFates [64], and MitoProt II [65]. Known nuclear proteins, mtDNA-encoded proteins, and nuclear DNA-encoded mitochondrial proteins were set respectively as negative and positive controls. However, none of the prediction tools used were able to identify functional MTS in the 5mC-associated proteins or cofactors, including DNMTs, TETs, UHRF1 and TDG (Fig. 6A). We then searched for 5mC-associated proteins in three mass-spectrometry-based mitochondrial proteomic datasets [66–69]. TFAM, CYC1, and FH, which are three canonical mitochondrial proteins, were used as positive controls. Consistent with the results of MTS prediction, mass spectrometry analysis did not identify DNMTs, TETs, or other co-factors in mitochondria (Fig. 6B). This suggests that either 5mC-associated proteins are not localized to the mitochondria or their abundance is too low to be detected, which is in tune with the absence of mtDNA 5mC.

## Discussion

**Fig. 7:**
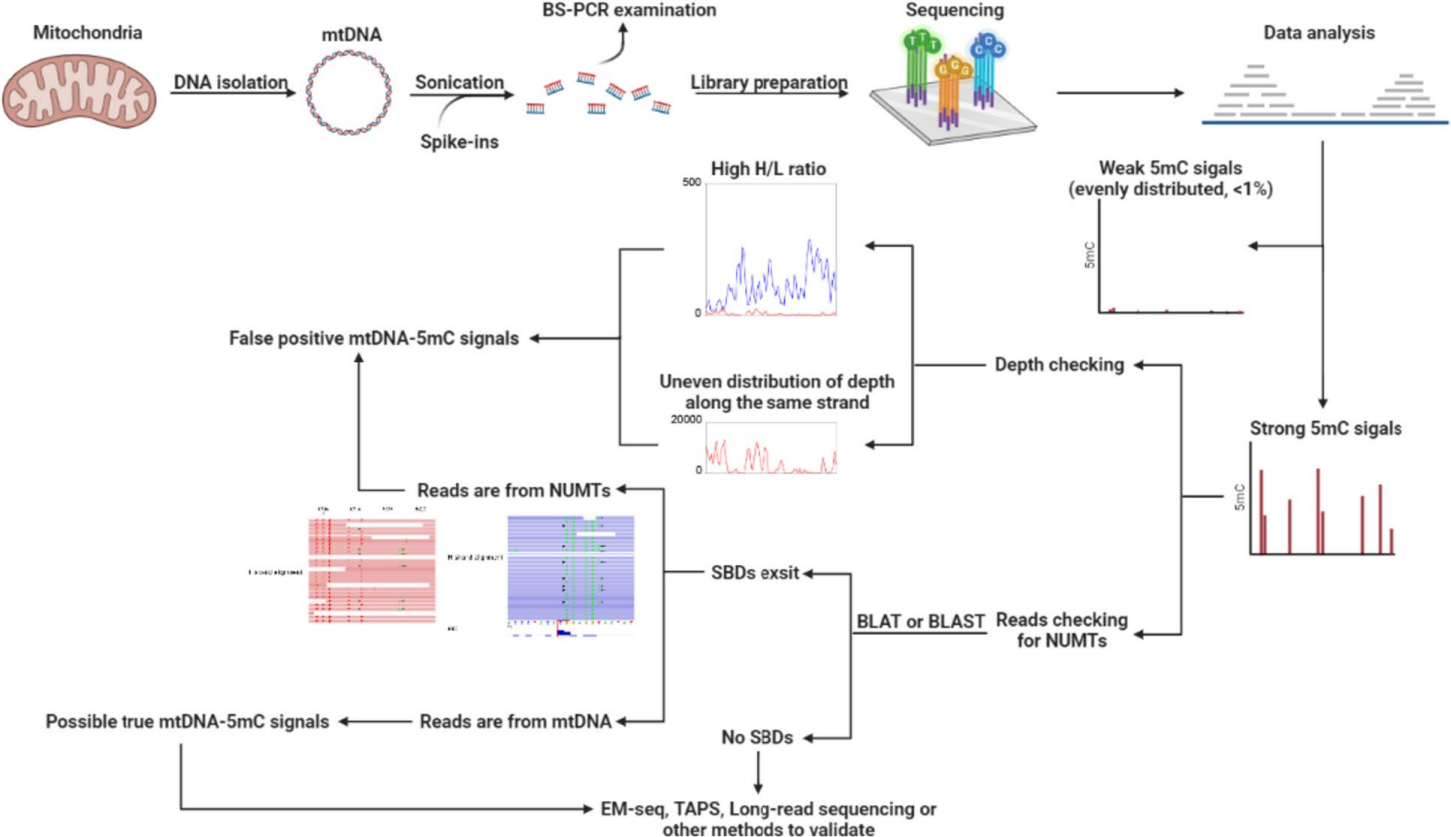
A framework to analyze mammalian mtDNA-5mC using WGBS. MtDNA should be enriched and fragmented by sonication, both of which help to bring down the false 5mC signals from NUMTs and provide more usable reads for mtDNA, before subjected to high-throughput sequencing. Sanger bisulfite sequencing is performed as quality control (forward primers without cytosines and reverse primers without guanines, or degenerate primers are recommended). For WGBS analysis, when strong 5mC signal (> 5%) appears, the sequencing depth should be compared with adjacent regions as well as the average depth of the entire strand in a strand-specific manner. Since the vast majority of cytosines in mtDNA was sequenced much deeper than those in nuclear DNA (depth cutoff was usually set as 5x or 10x), it is reasonable to examine the relative depth rather than the absolute depth when analyzing mtDNA-5mC. Meanwhile, the original sequences of the reads before C to T conversion should be subject to BLAT or BLAST to examine whether the 5mC signals are resulted from misaligned NUMTs reads. Because sequencing depth bias (both between strands and within strands) and reads from NUMTs are two main sources of mammalian mtDNA-5mC signals, quality control of reads distribution and examination of alignment are expected to filter false positive 5mC signals. Enzymatic conversion-based techniques, such as TAPS and EM-seq, largely eliminate the biases and artefacts observed in previous WGBS studies, making them promising options for future investigations

The report of 5mC in mammalian mitochondrial DNA in the 1970s has led to extensive studies aimed at quantifying and understanding the function of mtDNA-5mC. However, the existence and levels of mtDNA-5mC have been strongly questioned, and contradictory results have reported since then [31, 33–35, 43, 70]. Recently, it was reported that WGBS analysis revealed up to 40% methylation of cytosines in the mammalian mtDNA [16, 24]. Here, we show that a combination of factors, including insufficient bisulfite conversion rate, sequencing bias, and interference of NUMTs signals, contributed to the misidentification of 5mC in mammalian mitochondrial DNA. By combining our WGBS, TAPS, and EM-seq data, we reveal that the level of mtDNA-5mC is far lower (< 0.19 ~ 0.75%) than previously reported [16, 24], and falls below the detection limit of WGBS. In line with our findings, a recent critical reanalysis of the study by Patil et al. [16] put forward that the reported mtDNA-5mC signals were stemmed from a combination of methodological and technical pitfalls [70]. To identify reliable mtDNA-5mC signals using bisulfite sequencing, we propose a modified workflow (Fig. 7). This workflow involves enriching mtDNA to minimize interference from nuclear DNA, fragmenting mtDNA to avoid bias introduced by secondary structures during library preparation, and examining sequencing depths and SBDs of aligned reads at sites with high 5mC signals.

The interference from mtDNA secondary structures was not fully examined previously [22, 23, 25–29]. Several studies performed linearization of mtDNA prior to methylation analyses, however, the linearization efficiency was not evaluated [16, 48]. We show that linearization of mtDNA not only decreases but completely eliminates false positive 5mC signals in bisulfite PCR. This highlights the importance of evaluating the linearization efficiency before quantifying the 5mC content in mtDNA with bisulfite.

Since cytosines in the CpH context were reported to be methylated in mtDNA [16, 24], measurements of 5mC with classic Sanger bisulfite sequencing could be misleading due to amplification bias associated with non-degenerate primers. WGBS is an alternative approach to circumvent this problem. However, sequencing depth biases may still arise due to bisulfite-induced degradation [37] and the supercoiled structure of mtDNA [35]. Unmethylated C-rich fragments, which are not constrained by secondary structures, are susceptible to degradation after bisulfite reactions and therefore are absent from the library. Consequently, such libraries could be characterized with a severe inverse correlation between sequencing depth and mtDNA methylation level, as well as severe strand bias (high H/L ratio). Hence, we propose that for mtDNA methylation analysis using WGBS, it is crucial to carefully examine library quality in terms of sequencing depth and strand bias.

Another contributing factor to false positive signals of mtDNA-5mC in high-throughput sequencing is the tolerance of mismatches and the interference of NUMTs. In our study, we scrutinized the alignment file of the mouse sperm WGBS data and designed primers to distinguish between *Cox1* and NUMTs in *Tet* TKO mESC. Taking advantage of SBDs between mtDNA and NUMTs, we demonstrated that the detected 5mC signals in mouse sperm and *Tet* TKO mESC were erroneous detections caused by misaligned NUMTs reads. Our work indicates that enrichment of mtDNA is critical in reducing the false positive 5mC signals from NUMTs and therefore is highly recommended for mtDNA methylation analysis. For the convenience of future work, we provided representative mtDNA regions in which NUMTs contamination may affect methylation calling (Table S2 for mouse and Table S3 for human). It is essential to examine reads mapped to these regions to prevent overestimation of the methylation level of the mitochondrial genome.

DNA methylation usually presents together with histone modifications in eukaryotic cells, and the crosstalk between DNMTs and histone modifications is vital for mammalian cell activities. Common model organisms such as *Drosophila melanogaster*, *Caenorhabditis elegans*, fission yeast, and baker’s yeast possess histones but exhibit almost no 5mC [71]. The opposite case, with abundant DNA methylation but lacking histone modifications, is rarely reported in eukaryotic cells. Recent studies have reported the presence of some, but not the complete set of, DNA methylation machinery in mammalian mitochondria [16, 23, 24, 29, 59–61]. Apart from the discordances among these reports, the possibility of contamination from nuclear proteins cannot be entirely excluded. By analysing published mitochondrial databases and mass-spectrometry-based mitochondrial proteomic data, we found that 5mC-associated proteins and co-factors were absent in mitochondria across a variety of cells and tissues. Although DNMT1 has been reported to possess de novo methylation activity [72–74] and has been detected in mitochondria [59], it is worth noting that mtDNA is free of histones, which are essential for DNMT1 to maintain DNA methylation in vivo [71]. Therefore, it is unlikely for DNMT1 alone to produce extensive and functionally stable 5mC in mitochondria.

## Conclusions

In the present study, we provide evidence to suggest that the previous reported mtDNA-5mC are most likely resulted from inefficient bisulfite conversion, strand sequencing bias, and misaligned nuclear mitochondrial sequences. For the efficient development of this field, we propose an optimized bisulfite sequencing procedure, which mainly entails linearization of mtDNA for Sanger bisulfite sequencing, enrichment of mtDNA before WGBS, as well as carefully inspection of strand depth bias and NUMTs during the analysis workflow. With this framework in place, we show that 5mC in mtDNA is more likely a technical artifact rather than a relevant epigenetic mark. The maximum levels of 5mC observed in the analyzed cells and tissues are far lower than previously reported. Although it is not possible to completely exclude the existence of a few 5mC bases at levels below our detection limit (< 0.75%), and the potential for these bases to have biological functions, it is impractical to examine all types of cells under all conditions. Nevertheless, our results strongly imply that the published data are not sufficient to support the presence of 5mC in mammalian mtDNA, and the levels of 5mC are extremely low, provided the modification ever occurs.

## Materials and methods

### Cell lines

All cell lines were obtained from National Collection of Authenticated Cell Cultures (https://www.cellbank.org.cn/). Mouse embryonic stem cells (mESC) were cultured in DMEM (Gibco, 11965-092) supplemented with 15% FBS (Gibco, 30044-333), 1% penicillin-streptomycin (Gibco, 15140-122), 0.1 mM non-essential amino acids (Gibco, 11140-050), 2 mM L-glutamine (Gibco, 25030-081), 1 mM sodium pyruvate (Gibco, 11360-070), 0.1 mM β-mercaptoethanol, 1000 U/ml murine leukaemia inhibitory factor (LIF, Chemicon, ESG1107), 3 μM CHIR99021 (Selleck, S1263), 1 μM PD0325901 (Rodchem, L1002). Human lung carcinoma cell line A549 were cultured in α-MEM (Gibco, 12561-056) with 10% FBS (Gibco, 10270-106) and 1% penicillin-streptomycin. Human breast cancer cell line MCF7 were cultured in DMEM with 10% FBS (Gibco, 10270-106), 1% penicillin-streptomycin, 1 mM sodium pyruvate and 2 mM L-glutamine. All other cell lines were maintained in DMEM supplemented with 10% FBS (Gibco, 10270-106) and 1% penicillin-streptomycin. The cell culture incubator was set an atmosphere of 5% CO_2_ at 37 °C.

### Human subjects and platelets isolation

Human platelets were isolated from cord blood of healthy donors. Briefly, whole cord blood was added to the top of Ficoll reagent (GE Healthcare) and centrifuged at 500 × g for 30 min. The plasma layer containing platelets were transferred to a new tube and centrifuged at 3731 × g for 4 min to precipitate platelets [75].

### Oocyte micromanipulation

Metaphase II-arrested oocytes were obtained from adult female B6D2F1 mice by superovulation. After Hoechst 33342 staining, oocyte nuclei and polar bodies were removed by micromanipulations in a droplet of M2 medium (Millipore, MR-015-D) containing 5 μg/ml cytochalasin B (Sigma, C6762) using a blunt Piezo-driven pipette.

### DNA extraction

Total DNA was extracted from cells or tissues using Phenol-chloroform DNA extraction protocol.

### Linearization of mtDNA

Linearization of mouse and human mtDNA was achieved by FastDigest restriction endonuclease digestion. 500 ng mouse total DNA was digested with 2.5 μl *Sac*I (Thermo Scientific, FD1133) and 2.5 μl *Nco*I (Thermo Scientific, FD0573). 500 ng human total DNA was digested with 2.5 μl *Bbs*I (Thermo Scientific, FD1014) and 2.5 μl *Pvu*II (Thermo Scientific, FD0634). Linearization efficiency was analyzed on agarose gel and quantified by real-time qPCR with NovoStart SYBR qPCR SuperMix Plus (Novoprotein). Linearization efficiency was calculated based on the 2^−ΔΔCt^ method normalized by selected internal controls. Primers are listed in Table S4.

### Sanger bisulfite sequencing

Bisulfite conversion was performed using EZ DNA Methylation-Direct™ Kit (ZYMO Research) according to the manufacturer’s instructions. PCR products were cloned into T-vector using pClone007 Versatile Simple Vector Kit (TSINGKE) and individual clones were sequenced. Bisulfite sequencing results were analyzed using the online tool QUMA (www.quma.cdb.riken.jp/) [76]. Primers are listed in Table S5. Primers for regular PCR amplifications are listed in Table S6.

### MtDNA enrichment and efficiency analysis

Enrichment of mtDNA was performed using TIANprep Mini Plasmid Kit (TIANGEN) according to the manufacturer’s instructions. Relative enrichment of mtDNA over nuclear DNA was assessed based on the 2^−ΔΔCt^ method and normalized by selected internal controls. Primers are listed in Table S7.

### WGBS library preparation and data analysis of WGBS and EM-seq

Briefly, 200–1000 ng genomic DNA was used for WGBS library construction. DNA was sonicated to ~250 bp fragments with the Diagenode Bioruptor Pico. Then DNA was subjected to end-repair, A-tailing, and ligation using VAHTS Universal DNA Library Prep Kit for Illumina V3 (Vazyme, ND607) and methylated adaptor (Roche) according to the manufacturer’s instructions. DNA was purified with VAHTS DNA Clean Beads. Subsequently, library was bisulfite modified using QIAGEN EpiTect Fast DNA Bisulfite Kit (59824) and amplified with KAPA2G Robust HotStart PCR Kits with dNTPs (Sigma-Aldrich). Library was finally subjected to 150 bp paired-end sequencing using the Illumina Novaseq6000 instrument.

For data processing, raw data were trimmed by Trim Galore (v0.5.0) with default settings. Then, the clean data were mapped to the rCRS (NC_012920.1) or ChrM of mm10 mouse reference genome using Bismark (v0.20.1) [77] with the parameters -X 2000 --score_min L,0,-0.6 or BS-Seeker2 (v2.1.8) [78] with the parameter -m 3. Duplicates were removed using Picard (v2.22.4). Bigwig files were generated using bedGraphToBigWig (v4) and visualized in Integrative Genomics Viewer (IGV) [79]. Since we regarded sequencing depth as a relative concept and the vast majority of cytosines in mtDNA was sequenced far more than 50 times, we intentionally did not set a depth cutoff. For metaplot, sequencing depth were calculated for every 200-bp consecutive bins across the mitochondria genome (the first and the last 150 bp were ignored). Metaplots were generated using deepTools (v3.3.0) [80]. EM-seq was analyzed using the same method.

### TAPS library preparation and data processing

Whole-genome TAPS libraries were constructed as previously described [81]. For data processing, raw data were trimmed by Trim Galore (v0.5.0) with default settings. The clean data were mapped to ChrM of mm10 mouse reference genome using Bowtie2 (v2.2.5) [82]. Duplicates were removed using Picard (v2.22.4). Methylation levels were called using asTair (v 3.2.6) [51].

### MTS prediction

MTS was predicted in MitoMiner (v4.0) [83] database which integrates iPSORT, targetP, MitoFates and MitoProt II. The probability of human proteins was examined using the SEARCH function according to the website guidance.

### Mitochondrial proteomic analysis

High-confidence mitochondrial proteins were identified from three mass-spectrometry-based mitochondrial proteomic data, MitoCop, MitoCarta3.0 and APEX.

#### MitoCop

Nine hundred sixty-five human genes encoding proteins that satisfy the following filtering conditions according to spatial proteomics data from MitoCop were identified: 1) belong to cluster 1; 2) the sum of Mean Norm. MS Intensity cM and Mean Norm. MS Intensity pM is greater than 1.

#### MitoCarta3.0

All 1136 human genes encoding proteins with strong support of mitochondrial localization were identified by MitoCarta3.0.

##### APEX

The union of 495 human mitochondrial matrix proteins and 127 human mitochondrial intermembrane space proteins were identified by the ascorbate peroxidase (APEX) tagging method, with 33 overlapping proteins.

### Public datasets

Published datasets GSE92310, GSE127301, GSE87757, GSE56697 and GSE33722 were downloaded from Gene Expression Omnibus (GEO) and were summarized in Table S8. Data were processed as described above.

### Supplementary Information

**Additional file 1: Figure S1.** Low sequencing depth causes false methylation calls in the published WGBS data. **Figure S2.** WGBS mapping results of BSseeker2 are akin to those of Bismark. **Figure S3.** Methylation profiles of four HEK293T replicates. **Figure S4.** Methylation profiles of human cord blood-derived platelets. **Figure S5.** Methylation profiles of NA12878 DNA by WGBS and EM-seq. **Figure S6.** PCR amplification of mtDNA genes *Cox1*, *Cox2* and *Nd5 *in mouse sperm produces amplicons from NUMTs. **Figure S7.** No 5mC was detected in the mtDNA from mouse MII oocyte. **Figure S8.** The original image of uncropped DNA gel. **Table S1.** WGBS sequencing depth of L and H strand in different studies. **Table S2.** Representative NUMTs and corresponding regions in mtDNA in mouse genome (mm10 reference genome). **Table S3.** Representative NUMTs and corresponding regions in mtDNA in human genome (Hg38 reference genome). **Table S4.** Primers for linearization analysis. **Table S5.** PCR primers for Sanger Bisulfite sequencing. **Table S6.** Primers for PCR amplification of mtDNA genes. **Table S7.** qPCR primers for analyzing mtDNA enrichment. **Table S8.** Published datasets used in this study.

## Data Availability

All genomic sequencing data are available in Gene Expression Omnibus (GEO) database with the accession number GSE217117.

## References

[1] Ehrlich M, Gama-Sosa MA, Huang LH, Midgett RM, Kuo KC, McCune RA, Gehrke C (1982). Amount and distribution of 5-methylcytosine in human DNA from different types of tissues of cells. Nucleic Acids Res.

[2] Bird A (2002). DNA methylation patterns and epigenetic memory. Genes Dev.

[3] Weisenberger DJ, Campan M, Long TI, Kim M, Woods C, Fiala E, Ehrlich M, Laird PW (2005). Analysis of repetitive element DNA methylation by MethyLight. Nucleic Acids Res.

[4] Cedar H, Bergman Y (2012). Programming of DNA methylation patterns. Annu Rev Biochem.

[5] Smith ZD, Meissner A (2013). DNA methylation: roles in mammalian development. Nat Rev Genet.

[6] Falkenberg M, Larsson NG, Gustafsson CM (2007). DNA replication and transcription in mammalian mitochondria. Annu Rev Biochem.

[7] Gammage PA, Moraes CT, Minczuk M (2018). Mitochondrial genome engineering: the revolution may not be CRISPR-Ized. Trends Genet.

[8] Mokranjac D, Neupert W (2005). Protein import into mitochondria. Biochem Soc Trans.

[9] Garrido N, Griparic L, Jokitalo E, Wartiovaara J, van der Bliek AM, Spelbrink JN (2003). Composition and dynamics of human mitochondrial nucleoids. Mol Biol Cell.

[10] Kukat C, Davies KM, Wurm CA, Spahr H, Bonekamp NA, Kuhl I, Joos F, Polosa PL, Park CB, Posse V (2015). Cross-strand binding of TFAM to a single mtDNA molecule forms the mitochondrial nucleoid. Proc Natl Acad Sci U S A.

[11] Kukat C, Wurm CA, Spahr H, Falkenberg M, Larsson NG, Jakobs S (2011). Super-resolution microscopy reveals that mammalian mitochondrial nucleoids have a uniform size and frequently contain a single copy of mtDNA. Proc Natl Acad Sci U S A.

[12] Dzitoyeva S, Chen H, Manev H (2012). Effect of aging on 5-hydroxymethylcytosine in brain mitochondria. Neurobiol Aging.

[13] Pirola CJ, Gianotti TF, Burgueno AL, Rey-Funes M, Loidl CF, Mallardi P, Martino JS, Castano GO, Sookoian S (2013). Epigenetic modification of liver mitochondrial DNA is associated with histological severity of nonalcoholic fatty liver disease. Gut.

[14] Iacobazzi V, Castegna A, Infantino V, Andria G (2013). Mitochondrial DNA methylation as a next-generation biomarker and diagnostic tool. Mol Genet Metab.

[15] Blanch M, Mosquera JL, Ansoleaga B, Ferrer I, Barrachina M (2016). Altered mitochondrial DNA methylation pattern in Alzheimer disease-related pathology and in Parkinson disease. Am J Pathol.

[16] Patil V, Cuenin C, Chung F, Aguilera JRR, Fernandez-Jimenez N, Romero-Garmendia I, Bilbao JR, Cahais V, Rothwell J, Herceg Z (2019). Human mitochondrial DNA is extensively methylated in a non-CpG context. Nucleic Acids Res.

[17] Nass MM (1973). Differential methylation of mitochondrial and nuclear DNA in cultured mouse, hamster and virus-transformed hamster cells. In vivo and in vitro methylation. J Mol Biol.

[18] Pollack Y, Kasir J, Shemer R, Metzger S, Szyf M (1984). Methylation pattern of mouse mitochondrial DNA. Nucleic Acids Res.

[19] Shmookler Reis RJ, Goldstein S (1983). Mitochondrial DNA in mortal and immortal human cells. Genome number, integrity, and methylation. J Biol Chem.

[20] Infantino V, Castegna A, Iacobazzi F, Spera I, Scala I, Andria G, Iacobazzi V (2011). Impairment of methyl cycle affects mitochondrial methyl availability and glutathione level in Down’s syndrome. Mol Genet Metab.

[21] Ghosh S, Sengupta S, Scaria V (2014). Comparative analysis of human mitochondrial methylomes shows distinct patterns of epigenetic regulation in mitochondria. Mitochondrion.

[22] Bianchessi V, Vinci MC, Nigro P, Rizzi V, Farina F, Capogrossi MC, Pompilio G, Gualdi V, Lauri A (2016). Methylation profiling by bisulfite sequencing analysis of the mtDNA Non-Coding Region in replicative and senescent Endothelial Cells. Mitochondrion.

[23] Wong M, Gertz B, Chestnut BA, Martin LJ (2013). Mitochondrial DNMT3A and DNA methylation in skeletal muscle and CNS of transgenic mouse models of ALS. Front Cell Neurosci.

[24] Dou X, Boyd-Kirkup JD, McDermott J, Zhang X, Li F, Rong B, Zhang R, Miao B, Chen P, Cheng H (2019). The strand-biased mitochondrial DNA methylome and its regulation by DNMT3A. Genome Res.

[25] Rebelo AP, Williams SL, Moraes CT (2009). In vivo methylation of mtDNA reveals the dynamics of protein-mtDNA interactions. Nucleic Acids Res.

[26] van der Wijst MG, van Tilburg AY, Ruiters MH, Rots MG (2017). Experimental mitochondria-targeted DNA methylation identifies GpC methylation, not CpG methylation, as potential regulator of mitochondrial gene expression. Sci Rep.

[27] Byun HM, Panni T, Motta V, Hou L, Nordio F, Apostoli P, Bertazzi PA, Baccarelli AA (2013). Effects of airborne pollutants on mitochondrial DNA methylation. Part Fibre Toxicol.

[28] Janssen BG, Byun HM, Cox B, Gyselaers W, Izzi B, Baccarelli AA, Nawrot TS (2014). Variation of DNA methylation in candidate age-related targets on the mitochondrial-telomere axis in cord blood and placenta. Placenta.

[29] Bellizzi D, D’Aquila P, Scafone T, Giordano M, Riso V, Riccio A, Passarino G (2013). The control region of mitochondrial DNA shows an unusual CpG and non-CpG methylation pattern. DNA Res.

[30] Mposhi A, Van der Wijst MG, Faber KN, Rots MG (2017). Regulation of mitochondrial gene expression, the epigenetic enigma. Front Biosci (Landmark Ed).

[31] Dawid IB (1974). 5-methylcytidylic acid: absence from mitochondrial DNA of frogs and HeLa cells. Science.

[32] Matsuda S, Yasukawa T, Sakaguchi Y, Ichiyanagi K, Unoki M, Gotoh K, Fukuda K, Sasaki H, Suzuki T, Kang D (2018). Accurate estimation of 5-methylcytosine in mammalian mitochondrial DNA. Sci Rep.

[33] Hong EE, Okitsu CY, Smith AD, Hsieh CL (2013). Regionally specific and genome-wide analyses conclusively demonstrate the absence of CpG methylation in human mitochondrial DNA. Mol Cell Biol.

[34] Owa C, Poulin M, Yan L, Shioda T (2018). Technical adequacy of bisulfite sequencing and pyrosequencing for detection of mitochondrial DNA methylation: sources and avoidance of false-positive detection. PLoS One.

[35] Mechta M, Ingerslev LR, Fabre O, Picard M, Barres R (2017). Evidence suggesting absence of mitochondrial DNA methylation. Front Genet.

[36] Tanaka K, Okamoto A (2007). Degradation of DNA by bisulfite treatment. Bioorg Med Chem Lett.

[37] Olova N, Krueger F, Andrews S, Oxley D, Berrens RV, Branco MR, Reik W (2018). Comparison of whole-genome bisulfite sequencing library preparation strategies identifies sources of biases affecting DNA methylation data. Genome Biol.

[38] Lopez JV, Yuhki N, Masuda R, Modi W, O’Brien SJ (1994). Numt, a recent transfer and tandem amplification of mitochondrial DNA to the nuclear genome of the domestic cat. J Mol Evol.

[39] Maude H, Davidson M, Charitakis N, Diaz L, Bowers WHT, Gradovich E, Andrew T, Huntley D (2019). NUMT confounding biases mitochondrial heteroplasmy calls in favor of the reference allele. Front Cell Dev Biol.

[40] Aminuddin A, Ng PY, Leong CO, Chua EW (2020). Mitochondrial DNA alterations may influence the cisplatin responsiveness of oral squamous cell carcinoma. Sci Rep.

[41] Luth T, Wasner K, Klein C, Schaake S, Tse R, Pereira SL, Lass J, Sinkkonen L, Grunewald A, Trinh J (2021). Nanopore single-molecule sequencing for mitochondrial DNA methylation analysis: investigating Parkin-associated Parkinsonism as a proof of concept. Front Aging Neurosci.

[42] Goldsmith C, Rodriguez-Aguilera JR, El-Rifai I, Jarretier-Yuste A, Hervieu V, Raineteau O, Saintigny P, Chagoya de Sanchez V, Dante R, Ichim G (2021). Low biological fluctuation of mitochondrial CpG and non-CpG methylation at the single-molecule level. Sci Rep.

[43] Bicci I, Calabrese C, Golder ZJ, Gomez-Duran A, Chinnery PF (2021). Single-molecule mitochondrial DNA sequencing shows no evidence of CpG methylation in human cells and tissues. Nucleic Acids Res.

[44] Wang L, Zhang J, Duan J, Gao X, Zhu W, Lu X, Yang L, Zhang J, Li G, Ci W (2014). Programming and inheritance of parental DNA methylomes in mammals. Cell.

[45] Xie W, Barr CL, Kim A, Yue F, Lee AY, Eubanks J, Dempster EL, Ren B (2012). Base-resolution analyses of sequence and parent-of-origin dependent DNA methylation in the mouse genome. Cell.

[46] Kolesar JE, Wang CY, Taguchi YV, Chou SH, Kaufman BA (2013). Two-dimensional intact mitochondrial DNA agarose electrophoresis reveals the structural complexity of the mammalian mitochondrial genome. Nucleic Acids Res.

[47] Lin L, Liu Y, Xu F, Huang J, Daugaard TF, Petersen TS, Hansen B, Ye L, Zhou Q, Fang F (2018). Genome-wide determination of on-target and off-target characteristics for RNA-guided DNA methylation by dCas9 methyltransferases. Gigascience.

[48] Liu B, Du Q, Chen L, Fu G, Li S, Fu L, Zhang X, Ma C, Bin C (2016). CpG methylation patterns of human mitochondrial DNA. Sci Rep.

[49] Quispe-Tintaya W, White RR, Popov VN, Vijg J, Maslov AY (2013). Fast mitochondrial DNA isolation from mammalian cells for next-generation sequencing. Biotechniques.

[50] Melchinger H, Jain K, Tyagi T, Hwa J (2019). Role of platelet mitochondria: life in a nucleus-free zone. Front Cardiovasc Med.

[51] Liu Y, Siejka-Zielinska P, Velikova G, Bi Y, Yuan F, Tomkova M, Bai C, Chen L, Schuster-Bockler B, Song CX (2019). Bisulfite-free direct detection of 5-methylcytosine and 5-hydroxymethylcytosine at base resolution. Nat Biotechnol.

[52] Vaisvila R, Ponnaluri VKC, Sun Z, Langhorst BW, Saleh L, Guan S, Dai N, Campbell MA, Sexton BS, Marks K (2021). Enzymatic methyl sequencing detects DNA methylation at single-base resolution from picograms of DNA. Genome Res.

[53] Wei W, Schon KR, Elgar G, Orioli A, Tanguy M, Giess A, Tischkowitz M, Caulfield MJ, Chinnery PF (2022). Nuclear-embedded mitochondrial DNA sequences in 66,083 human genomes. Nature.

[54] Hazkani-Covo E, Zeller RM, Martin W (2010). Molecular poltergeists: mitochondrial DNA copies (numts) in sequenced nuclear genomes. PLoS Genet.

[55] Reznik E, Miller ML, Senbabaoglu Y, Riaz N, Sarungbam J, Tickoo SK, Al-Ahmadie HA, Lee W, Seshan VE, Hakimi AA (2016). Mitochondrial DNA copy number variation across human cancers. Elife.

[56] Lightowlers RN, Chinnery PF, Turnbull DM, Howell N (1997). Mammalian mitochondrial genetics: heredity, heteroplasmy and disease. Trends Genet.

[57] Wai T, Ao A, Zhang X, Cyr D, Dufort D, Shoubridge EA (2010). The role of mitochondrial DNA copy number in mammalian fertility. Biol Reprod.

[58] May-Panloup P, Chretien MF, Savagner F, Vasseur C, Jean M, Malthiery Y, Reynier P (2003). Increased sperm mitochondrial DNA content in male infertility. Hum Reprod.

[59] Shock LS, Thakkar PV, Peterson EJ, Moran RG, Taylor SM (2011). DNA methyltransferase 1, cytosine methylation, and cytosine hydroxymethylation in mammalian mitochondria. Proc Natl Acad Sci U S A.

[60] Chestnut BA, Chang Q, Price A, Lesuisse C, Wong M, Martin LJ (2011). Epigenetic regulation of motor neuron cell death through DNA methylation. J Neurosci.

[61] Saini SK, Mangalhara KC, Prakasam G, Bamezai RNK (2017). DNA Methyltransferase1 (DNMT1) Isoform3 methylates mitochondrial genome and modulates its biology. Sci Rep.

[62] Bannai H, Tamada Y, Maruyama O, Nakai K, Miyano S (2002). Extensive feature detection of N-terminal protein sorting signals. Bioinformatics.

[63] Emanuelsson O, Nielsen H, Brunak S, von Heijne G (2000). Predicting subcellular localization of proteins based on their N-terminal amino acid sequence. J Mol Biol.

[64] Fukasawa Y, Tsuji J, Fu SC, Tomii K, Horton P, Imai K (2015). MitoFates: improved prediction of mitochondrial targeting sequences and their cleavage sites. Mol Cell Proteomics.

[65] Claros MG, Vincens P (1996). Computational method to predict mitochondrially imported proteins and their targeting sequences. Eur J Biochem.

[66] Morgenstern M, Peikert CD, Lubbert P, Suppanz I, Klemm C, Alka O, Steiert C, Naumenko N, Schendzielorz A, Melchionda L (2021). Quantitative high-confidence human mitochondrial proteome and its dynamics in cellular context. Cell Metab.

[67] Rath S, Sharma R, Gupta R, Ast T, Chan C, Durham TJ, Goodman RP, Grabarek Z, Haas ME, Hung WHW (2021). MitoCarta3.0: an updated mitochondrial proteome now with sub-organelle localization and pathway annotations. Nucleic Acids Res.

[68] Rhee HW, Zou P, Udeshi ND, Martell JD, Mootha VK, Carr SA, Ting AY (2013). Proteomic mapping of mitochondria in living cells via spatially restricted enzymatic tagging. Science.

[69] Hung V, Zou P, Rhee HW, Udeshi ND, Cracan V, Svinkina T, Carr SA, Mootha VK, Ting AY (2014). Proteomic mapping of the human mitochondrial intermembrane space in live cells via ratiometric APEX tagging. Mol Cell.

[70] Guitton R, Nido GS, Tzoulis C (2022). No evidence of extensive non-CpG methylation in mtDNA. Nucleic Acids Res.

[71] Greenberg MVC, Bourc’his D (2019). The diverse roles of DNA methylation in mammalian development and disease. Nat Rev Mol Cell Biol.

[72] Li Y, Zhang Z, Chen J, Liu W, Lai W, Liu B, Li X, Liu L, Xu S, Dong Q (2018). Stella safeguards the oocyte methylome by preventing de novo methylation mediated by DNMT1. Nature.

[73] Wang Q, Yu G, Ming X, Xia W, Xu X, Zhang Y, Zhang W, Li Y, Huang C, Xie H (2020). Imprecise DNMT1 activity coupled with neighbor-guided correction enables robust yet flexible epigenetic inheritance. Nat Genet.

[74] Haggerty C, Kretzmer H, Riemenschneider C, Kumar AS, Mattei AL, Bailly N, Gottfreund J, Giesselmann P, Weigert R, Brandl B (2021). Dnmt1 has de novo activity targeted to transposable elements. Nat Struct Mol Biol.

[75] Kahn RA, Cossette I, Friedman LI (1976). Optimum centrifugation conditions for the preparation of platelet and plasma products. Transfusion.

[76] Kumaki Y, Oda M, Okano M (2008). QUMA: quantification tool for methylation analysis. Nucleic Acids Res.

[77] Krueger F, Andrews SR (2011). Bismark: a flexible aligner and methylation caller for Bisulfite-Seq applications. Bioinformatics.

[78] Guo W, Fiziev P, Yan W, Cokus S, Sun X, Zhang MQ, Chen PY, Pellegrini M (2013). BS-Seeker2: a versatile aligning pipeline for bisulfite sequencing data. BMC Genomics.

[79] Thorvaldsdottir H, Robinson JT, Mesirov JP (2013). Integrative Genomics Viewer (IGV): high-performance genomics data visualization and exploration. Brief Bioinform.

[80] Ramirez F, Ryan DP, Gruning B, Bhardwaj V, Kilpert F, Richter AS, Heyne S, Dundar F, Manke T (2016). deepTools2: a next generation web server for deep-sequencing data analysis. Nucleic Acids Res.

[81] Xu Q, Wang C, Zhou JX, Xu ZM, Gao J, Sui P, Walsh CP, Ji H, Xu GL (2022). Loss of TET reprograms Wnt signaling through impaired demethylation to promote lung cancer development. Proc Natl Acad Sci U S A.

[82] Langmead B, Salzberg SL (2012). Fast gapped-read alignment with Bowtie 2. Nat Methods.

[83] Smith AC, Robinson AJ (2019). MitoMiner v4.0: an updated database of mitochondrial localization evidence, phenotypes and diseases. Nucleic Acids Res.

