# Supplementary material for: Optimized bisulfite sequencing analysis reveals the lack of 5-methylcytosine in mammalian mitochondrial DNA
Source: BMC Genomics. 2023 Aug 4;24:439. doi: 10.1186/s12864-023-09541-9 (PMC10403921; doi:10.1186/s12864-023-09541-9)
Supplement: Supplementary file 1 — Additional file 1: Figure S1. Low sequencing depth causes false methylation calls in the published WGBS data. Figure S2. WGBS mapping results of BSseeker2 are akin to those of Bismark. Figure S3. Methylation profiles of four HEK293T replicates. Figure S4. Methylation profiles of human cord blood-derived platelets. Figure S5. Methylation profiles of NA12878 DNA by WGBS and EM-seq. Figure S6. PCR amplification of mtDNA genes Cox1, Cox2 and Nd5 in mouse sperm produces amplicons from NUMTs. Figure S7. No 5mC was detected in the mtDNA from mouse MII oocyte. Figure S8. The original image of uncropped DNA gel. Table S1. WGBS sequencing depth of L and H strand in different studies. Table S2. Representative NUMTs and corresponding regions in mtDNA in mouse genome (mm10 reference genome). Table S3. Representative NUMTs and corresponding regions in mtDNA in human genome (Hg38 reference genome). Table S4. Primers for linearization analysis. Table S5. PCR primers for Sanger Bisulfite sequencing. Table S6. Primers for PCR amplification of mtDNA genes. Table S7. qPCR primers for analyzing mtDNA enrichment. Table S8. Published datasets used in this study. [file 12864_2023_9541_MOESM1_ESM.docx]

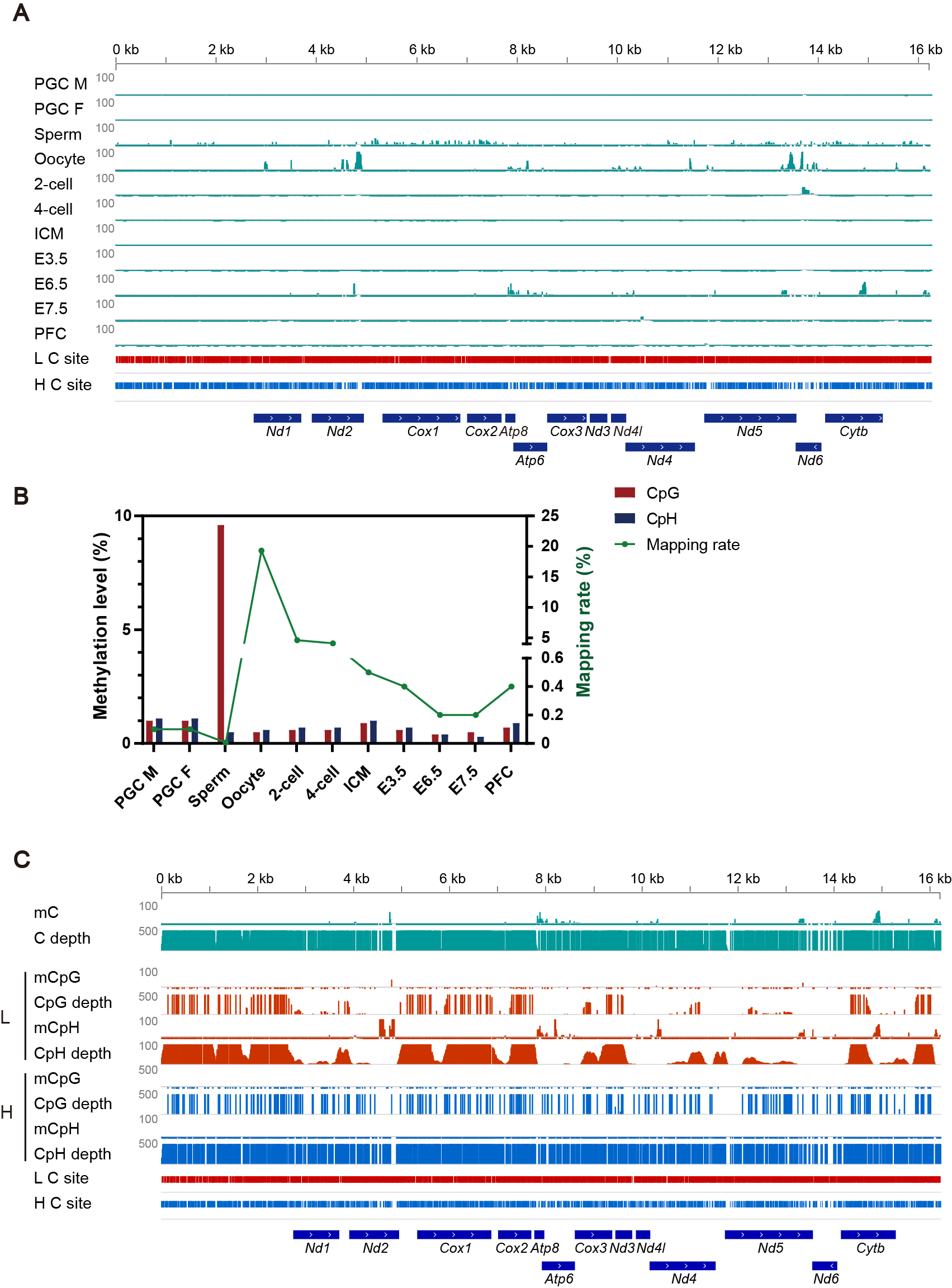


**Figure S1. Low sequencing depth causes false methylation calls in the published WGBS data.**

**(A)** Re-analysis of published WGBS datasets comprising mouse primordial germ cells (PGCs, male and female), sperm, oocyte, early embryos at 2-cell, 4-cell, ICM, E3.5, E6.5, E7.5 stages (GSE56697) and mouse prefrontal cortex (PFC) (GSM830249). 5mC signals were observed in the mtDNA of sperm, oocyte and E6.5 embryo. Methylation tracks scale from 0 to 100. Cytosine sites in each strand were marked on the bottom.

**(B)** Mean methylation levels of CpG and CpH sites (left y-axis) and mapping rates (green line, right y-axis) for mtDNA derived from indicated cell and tissue types.

**(C)** L strand-specific 5mC signals were detected in the mtDNA of early embryo at E6.5 (GSE56697). Methylation levels and sequencing depths of CpG and CpH were shown separately for L (red tracks) and R (blue tracks) strand. Methylation tracks scale from 0 to 100, and depth tracks scale from 0 to 500. 5mC signals present at CpH sites with extremely low sequencing depth in the L strand.


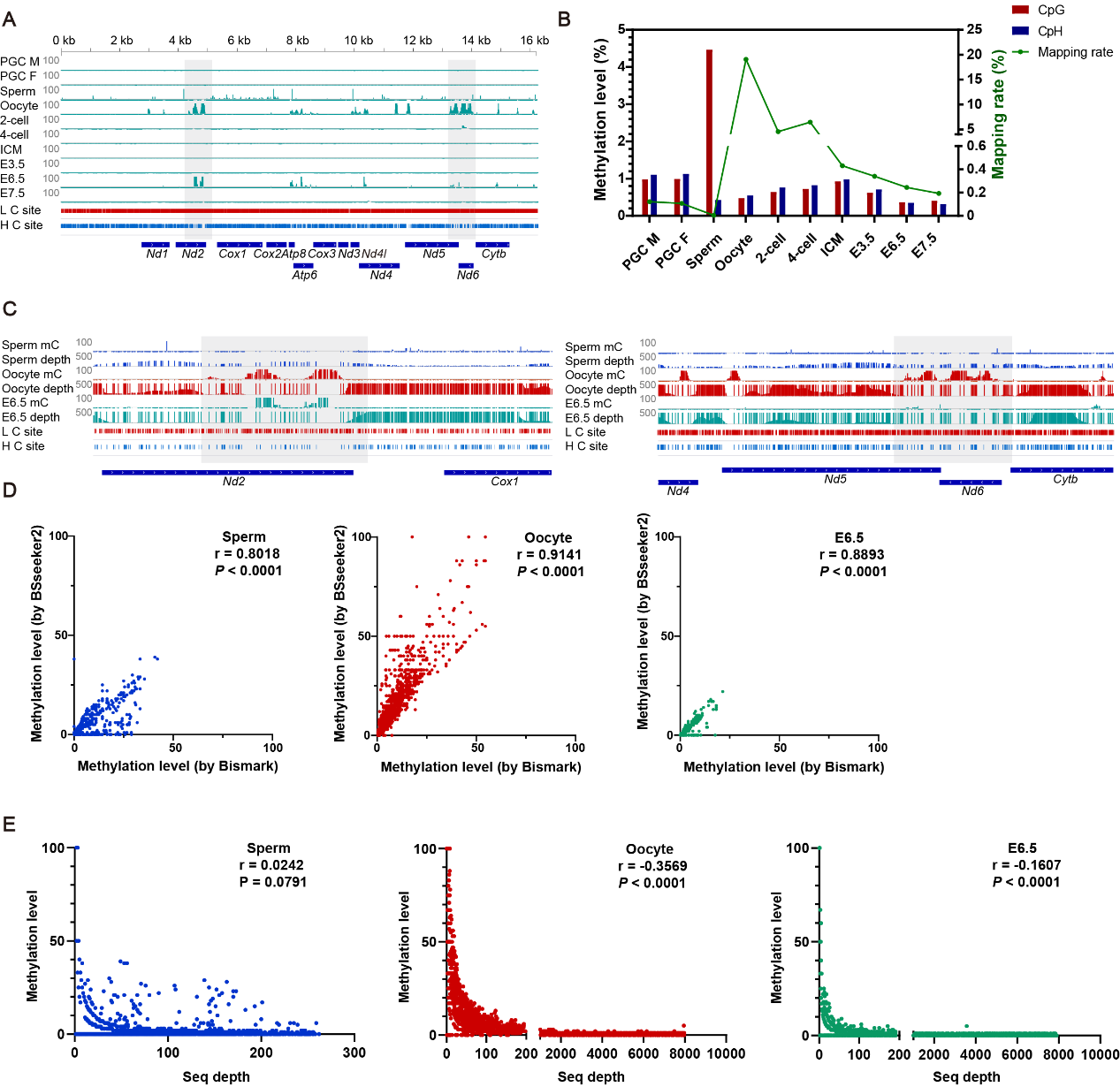


**Figure S2. WGBS mapping results of BSseeker2 are akin to those of Bismark.**

**(A)** Methylation profiles of mtDNA in mouse male and female PGC, sperm, oocyte and early embryos at 2-cell, 4-cell, ICM, E3.5, E6.5, E7.5 stages. WGBS datasets (GSE56697) were mapped with BSseeker2. 5mC signals were detected in the mtDNA of sperm, oocyte and E6.5 embryo.

**(B)** Mean mtDNA methylation levels of CpG and CpH sites (left y-axis) and mapping rates (green line, right y-axis) for indicated cell and tissue types.

**(C)** Positive 5mC signals were detected in the mtDNA of sperm, oocyte and E6.5 embryo at regions with low sequencing depth.

**(D)** Linear correlation between mtDNA methylation levels called by Bismark and BSseeker2. Each dot refers to one cytosine site in mtDNA.

**(E)** Inverse correlation between sequencing depth and methylation level. Each dot refers to one cytosine site in mtDNA.


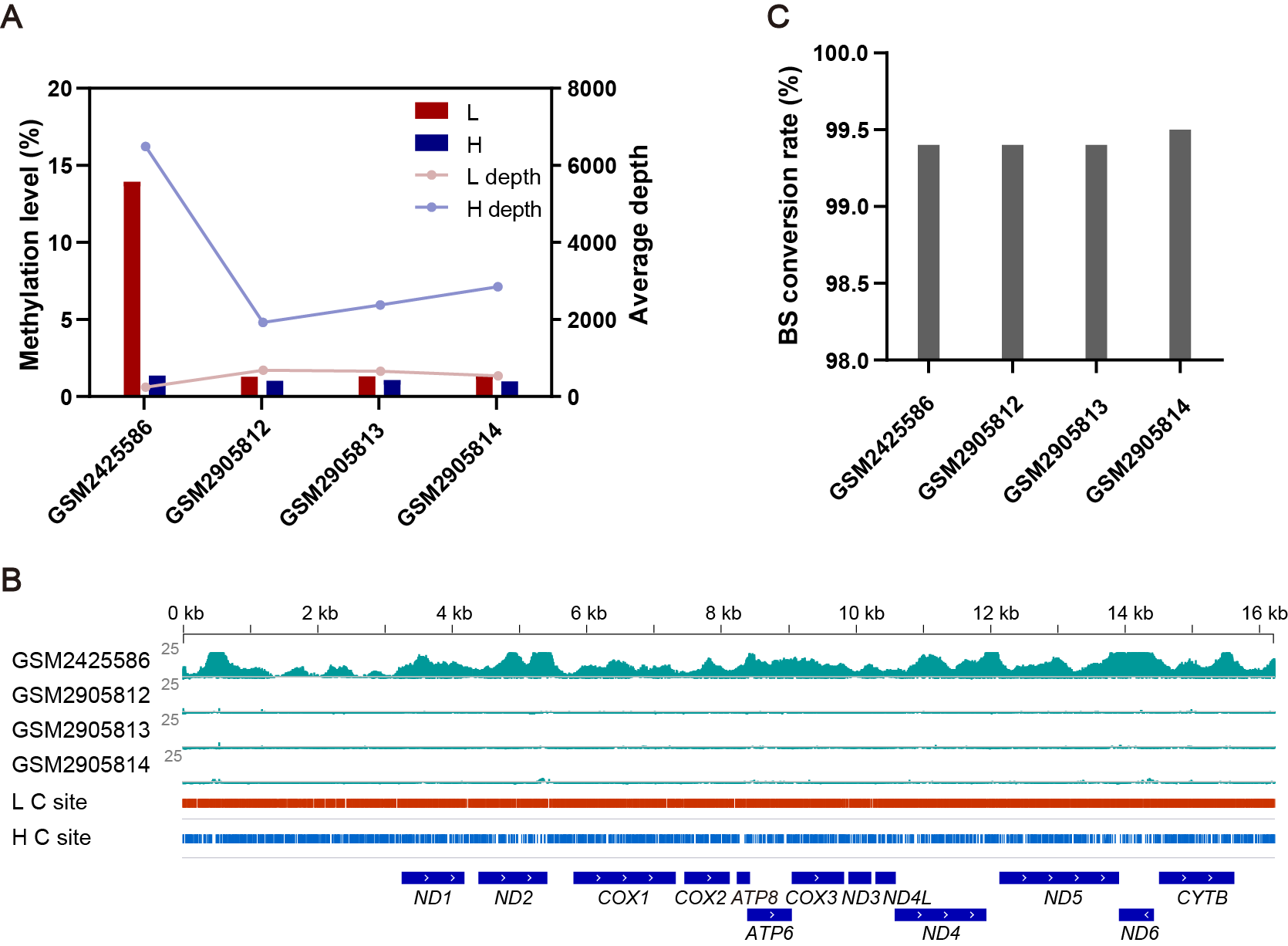


**Figure S3. Methylation profiles of four HEK293T replicates.**

**(A)** Mean methylation levels of L and H strand (left y-axis) and sequencing depths for L (pink line) and H (blue line) (right y-axis) strand for indicated datasets.

**(B)** Methylation profiles of mtDNA in HEK293T cell lines vary substantially among biological replicates in the data series of GSE92310.

**(C)** Bisulfite conversion rates of each HEK293T genomic DNA replicate.


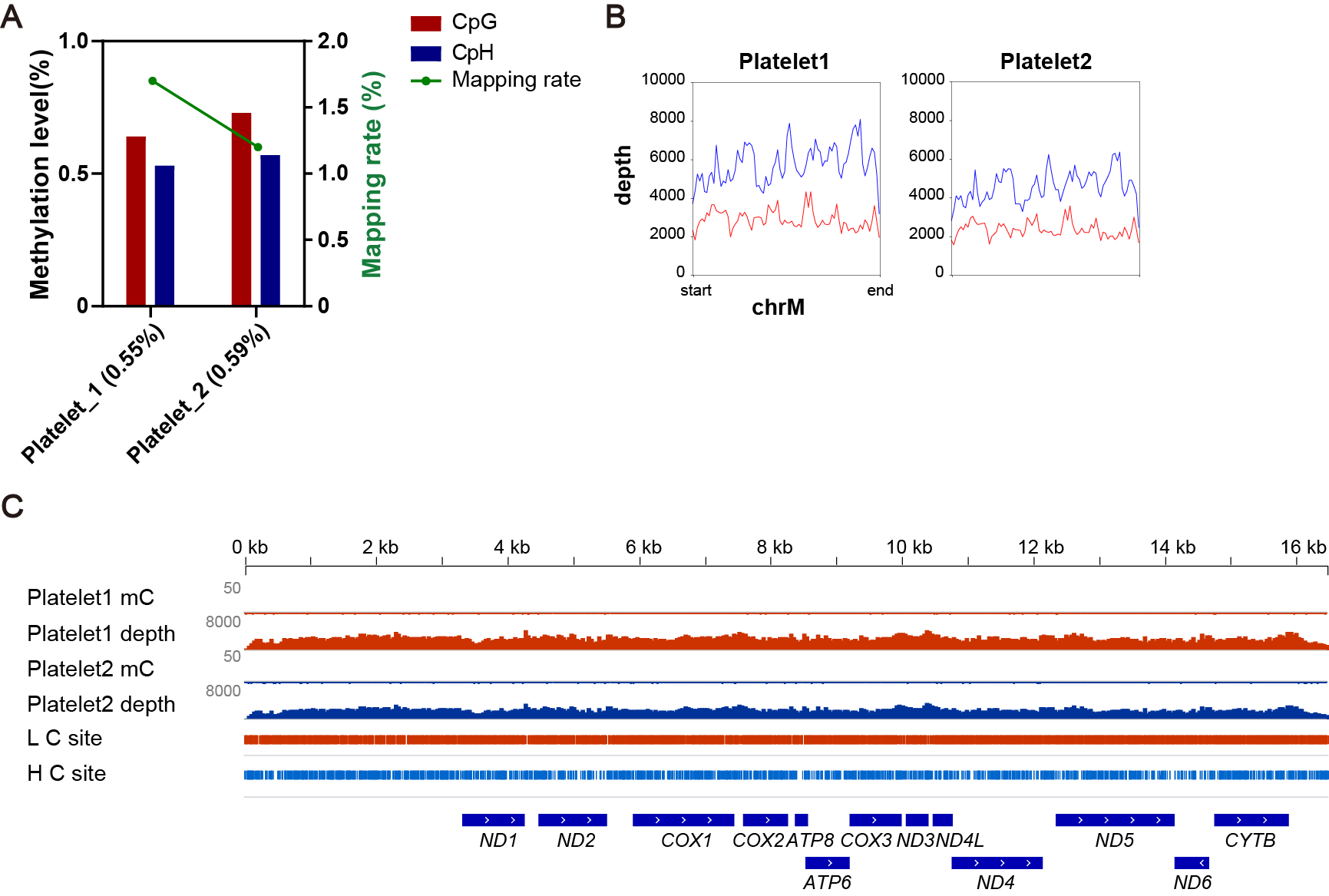
 **Figure S4. Methylation profiles of human cord blood-derived platelets.**

**(A)** Bar graph showing CpG and CpH methylation levels (left y-axis) and mapping rates (green line, right y-axis) for platelets isolated from two different cord blood donors. Average methylation levels are shown in the parentheses.

**(B)** Metaplots for the sequencing depths across the mtDNA of platelets.

**(C)** Methylation levels and sequencing depths of mtDNA in platelets measured by WGBS.

**
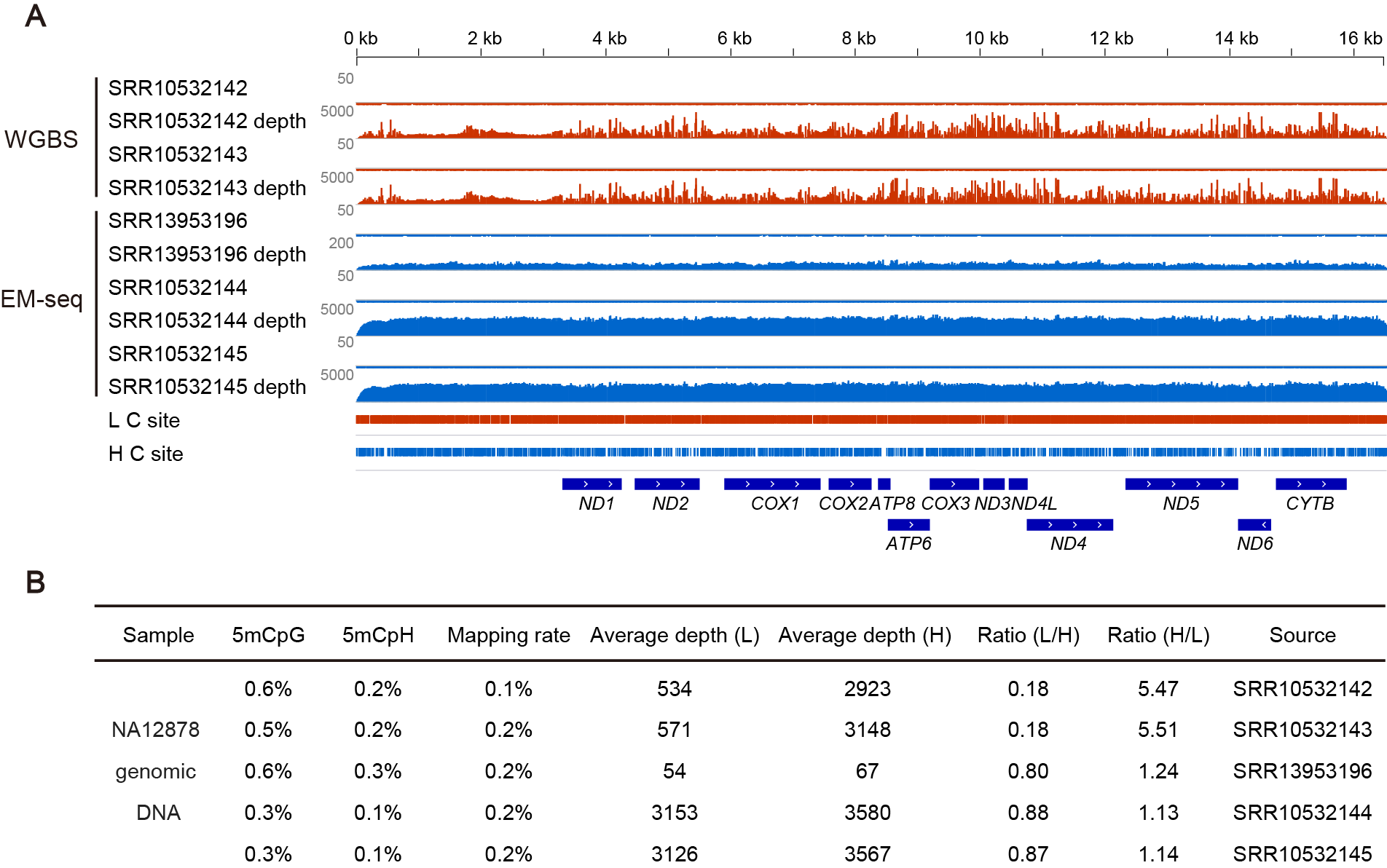
**

**Figure S5. Methylation profiles of NA12878 DNA by WGBS and EM-seq.**

**(A)** Methylation levels and sequencing depths of mtDNA measured by EM-seq and WGBS. EM-seq provided evenly distributed reads across the mtDNA.

**(B)** Mean mtDNA methylation levels of CpG and CpH sites, mapping rates, and sequencing depth of L and H strands.


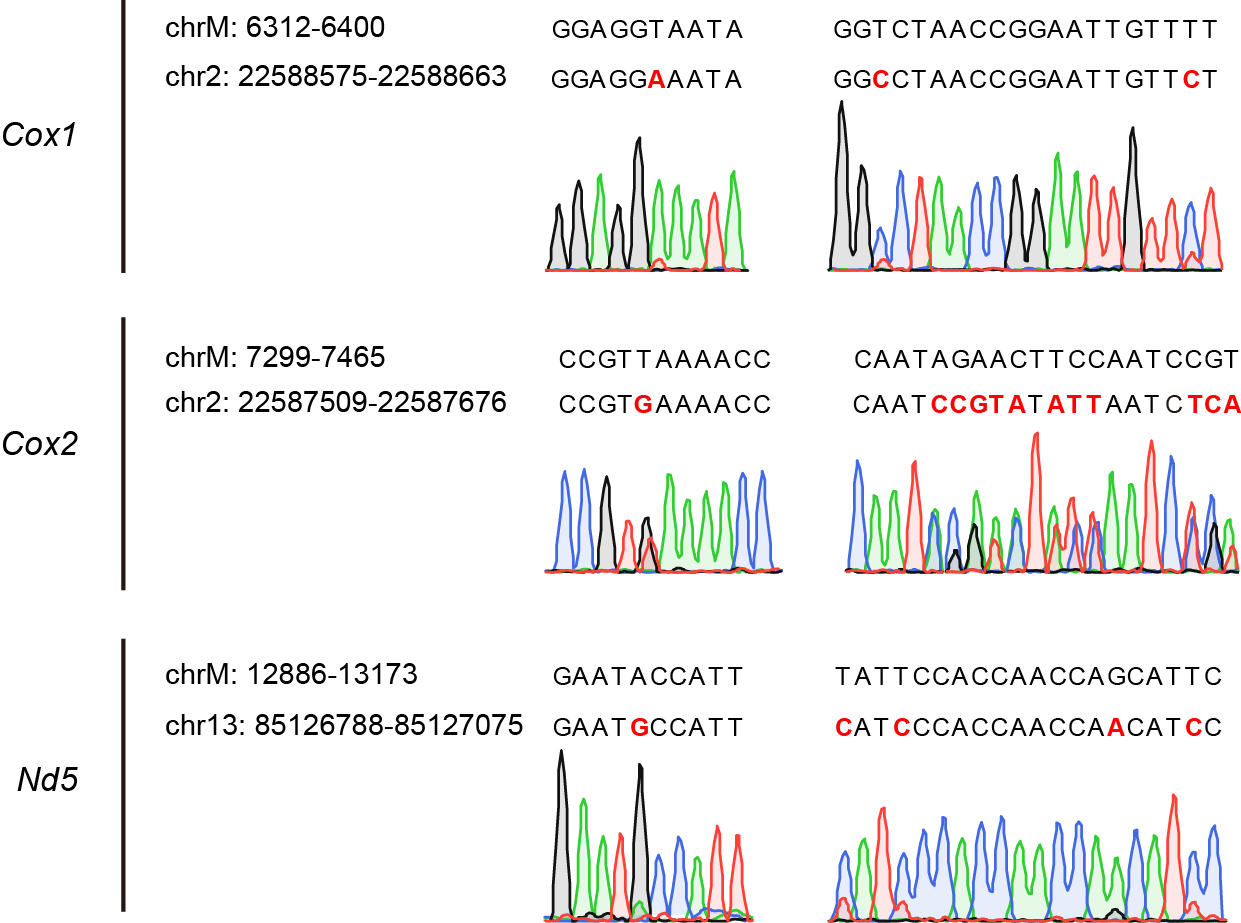


**Figure S6. PCR amplification of mtDNA genes *Cox1*, *Cox2* and *Nd5* in mouse sperm produces amplicons from NUMTs.**

Sanger sequencing towards *Cox1*, *Cox2* and *Nd5*. Double peaks in the chromatograms suggest a mixed pool of mtDNA and NUMTs amplicons. SBDs between mtDNA and NUMTs are shown in red font.


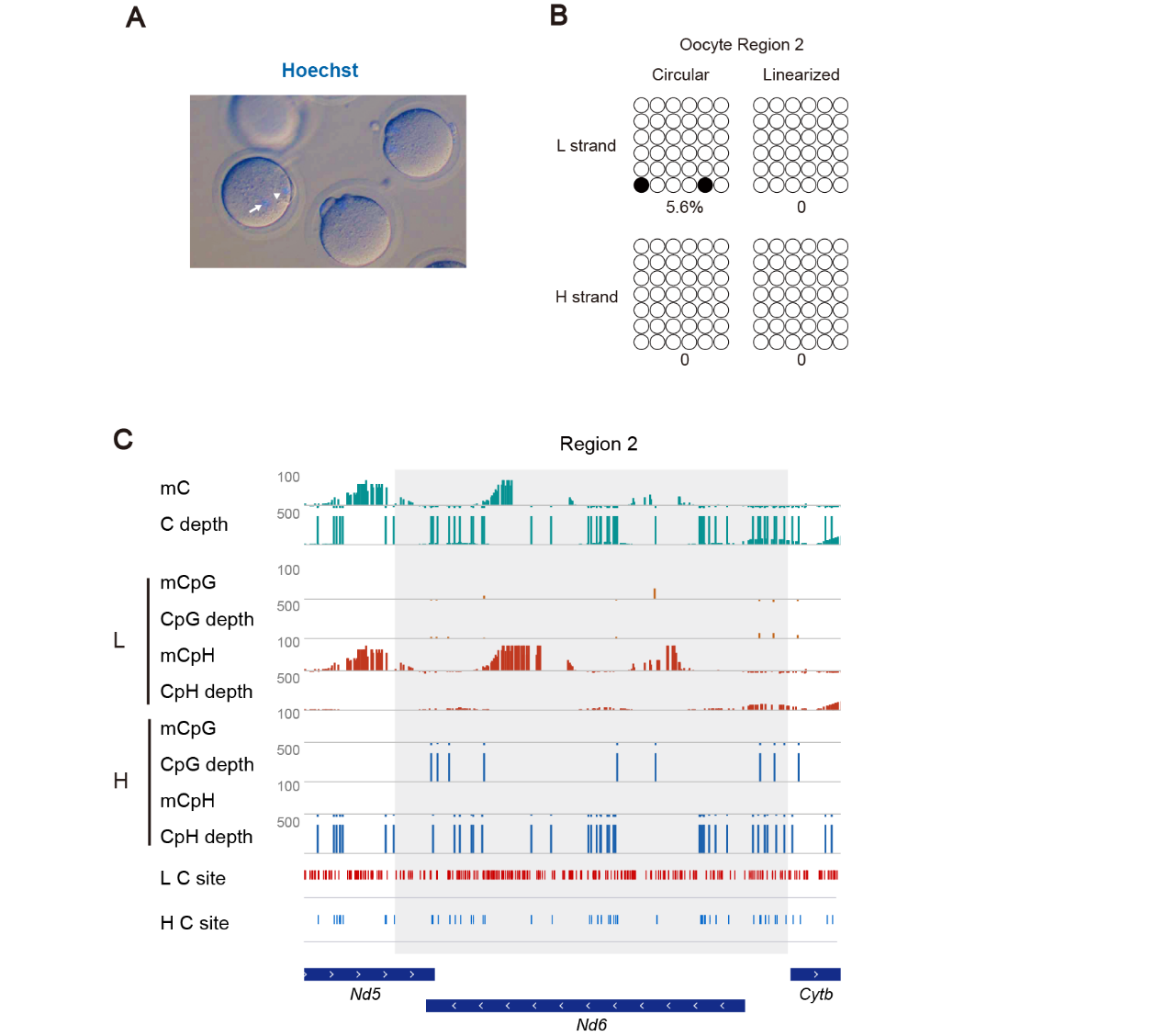


**Figure S7. No 5mC was detected in the mtDNA from mouse MII oocyte.**

**(A)** Hoechst staining image of MII oocytes. Arrow, nuclear DNA. Arrowhead, polar body DNA.

**(B)** Methylation analysis of MII oocyte mtDNA. Selected regions (chrM: 13483-14002 for L strand and chrM: 13440-13993 for H strand) within *Nd6* gene locus were examined by Sanger bisulfite sequencing with or without linearization of mtDNA. Oocyte nucleus and polar body were removed by micromanipulations to avoid interfering signals from NUMTs. Open and filled circles represent unmethylated and methylated CpG sites, respectively.

**(C)** A close-up view of Region 2. Methylation tracks scale from 0 to 100, and depth tracks scale from 0 to 500. Coverage ≥ 3 for 5mC, and ≥ 1 for mCpG and mCpH on each strand.


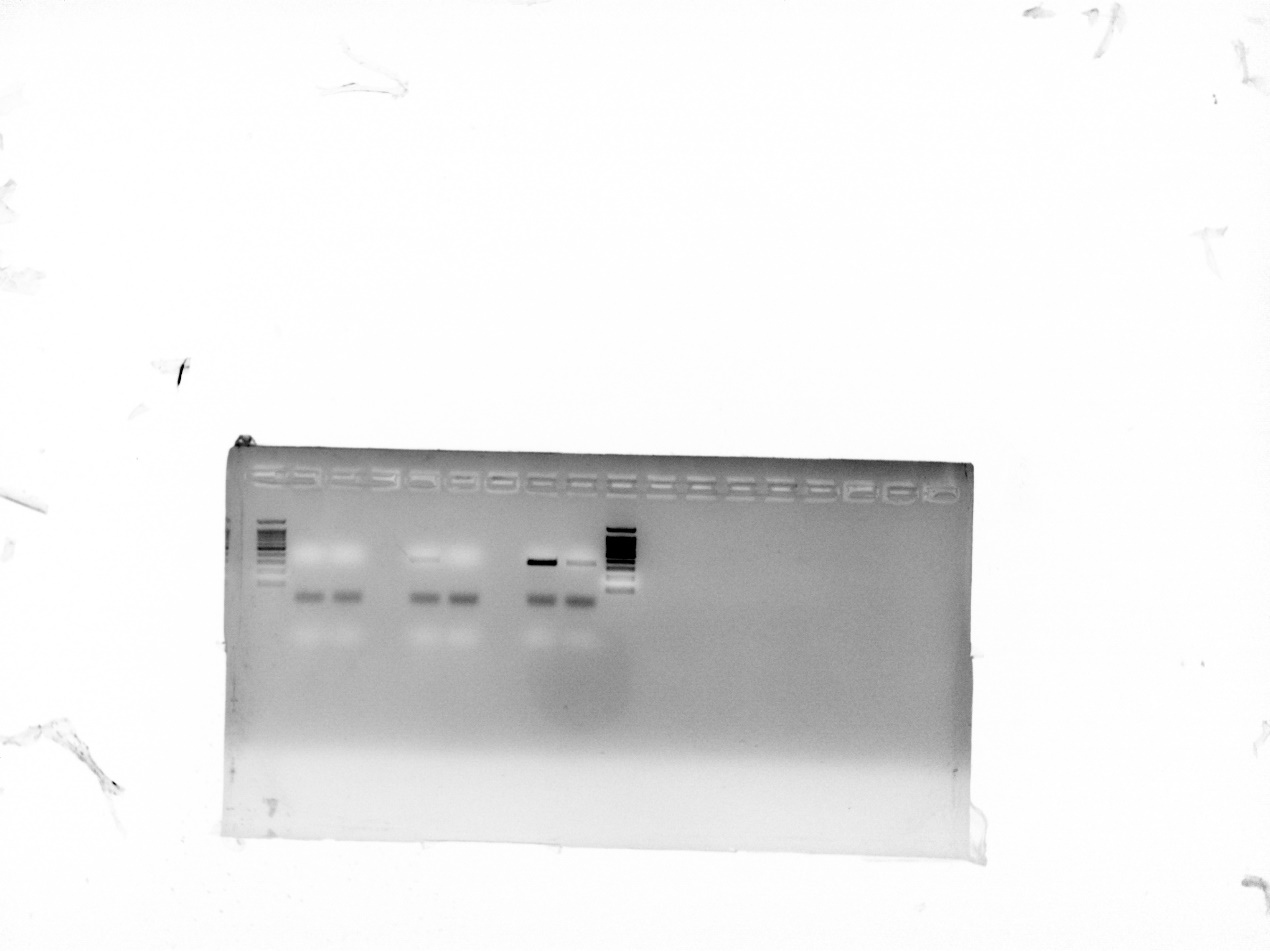
**Figure S8. The original image of uncropped DNA gel.**

PCR analysis of the linearization efficiency of mtDNA from N2a cells using primers flanking the cutting sites.

**Table S1. WGBS sequencing depth of L and H strand in different studies.**

| Sample | Average depth (L) | Average depth (H) | Ratio (L/H) | Ratio (H/L) | Source | |
| --- | --- | --- | --- | --- | --- | --- |
| HEK293T | 174 | 237 | 0.73 | 1.36 | This study |  |
| MCF7 | 98 | 139 | 0.71 | 1.42 |  |  |
| HepG2 | 418 | 604 | 0.69 | 1.44 |  |  |
| HepG2_P | 10557 | 11624 | 0.91 | 1.10 |  |  |
| N2a | 121 | 156 | 0.78 | 1.29 |  |  |
| N2a_P | 3121 | 3296 | 0.95 | 1.06 |  |  |
| mouse brain | 160 | 202 | 0.79 | 1.26 |  |  |
| mESC | 141 | 167 | 0.84 | 1.18 |  |  |
| *Tet* TKO mESC | 145 | 178 | 0.81 | 1.23 |  |  |
| B16 | 1359 | 1429 | 0.95 | 1.05 |  |  |
| LUAD cell line | 243 | 441 | 0.55 | 1.81 |  |  |
| platelet_1 | 2801 | 5443 | 0.51 | 1.94 |  |  |
| platelet_2 | 2297 | 4336 | 0.53 | 1.89 |  |  |
| HEK293T | 244 | 6489 | 0.04 | 26.59 | GSM2425586 |  |
| HEK293T | 686 | 1923 | 0.36 | 2.80 | GSM2905812 |  |
| HEK293T | 656 | 2377 | 0.28 | 3.62 | GSM2905813 |  |
| HEK293T | 535 | 2853 | 0.19 | 5.33 | GSM2905814 |  |
| A549 | 2266 | 8405 | 0.27 | 3.71 | GSM3633944 |  |
| mESC | 1658 | 2378 | 0.70 | 1.43 | GSM2339908 |  |
| mouse sperm | 8 | 101 | 0.08 | 12.63 | GSE56697 |  |
| mouse E6.5 | 427 | 5412 | 0.08 | 12.67 | GSE56697 |  |
| mouse oocyte | 2752 | 24141 | 0.11 | 8.77 | GSE56697 |  |

**Table S2. Representative NUMTs and corresponding regions in mtDNA in mouse genome (mm10 reference genome).**

| chrM | Main NUMTs | Strand | Length | Match |
| --- | --- | --- | --- | --- |
| chrM:58-454 | chr14:37135633-37136022 | - | 396 | 297 |
| chrM:1511-1881 | chr10:96077524-96077894 | - | 370 | 362 |
| chrM:1965-2734 | chr9:15319138-15319840 | + | 769 | 468 |
| chrM:3277-3915 | chr4:9958701-9959352 | + | 638 | 499 |
| chrM:3395-3816 | chr12:59262676-59263096 | + | 421 | 364 |
| chrM:4440-7699 | chr2:22587287-22590534 | - | 3259 | 3174 |
| chrM:6393-11042 | chr1:24611534-24616184 | - | 4649 | 4648 |
| chrM:6580-9390 | chr9:93173017-93175957 | - | 2810 | 1699 |
| chrM:12444-13400 | chr13:85126561-85127515 | - | 956 | 909 |
| chrM:12486-14284 | chr4:80002329-80004128 | + | 1798 | 1667 |
| chrM:13240-15356 | chr4:80003083-80005200 | + | 2116 | 1964 |
| chrM:14747-15714 | chr5:7276333-7277300 | + | 967 | 644 |
| chrM:15422-16054 | chr1:132790893-132791358 | + | 632 | 425 |

**Table S3. Representative NUMTs and corresponding regions in mtDNA in human genome (Hg38 reference genome).**

| chrM | Main NUMTs | Strand | Length | Match |
| --- | --- | --- | --- | --- |
| chrM:339-2697 | chr5:80650021-80652368 | - | 2358 | 2175 |
| chrM:519-2720 | chr11:10508138-10510336 | - | 2201 | 2083 |
| chrM:2225-10322 | chr17:22523654-22531725 | + | 8096 | 6410 |
| chrM:3912-9755 | chr1:629083-634924 | + | 5843 | 5758 |
| chrM:6115-12939 | chr5:100048182-100055045 | - | 6824 | 5988 |
| chrM:10267-15370 | chr5:134923425-134928527 | - | 5103 | 4801 |
| chrM:12660-16124 | chr5:94567455-94570918 | + | 3464 | 2988 |
| chrM:15167-16501 | chr4:64607082-64608409 | - | 1334 | 849 |
| chrM:15513-15909 | chr17:22520419-22520738 | + | 396 | 256 |

**Table S4. Primers for linearization analysis.**

| **Method** | **Targets** | **Primer sequence (5’-3’)** |
| --- | --- | --- |
| PCR and Agarose gel | Cutting sites | F-TCCCACTACTTAATACTTCAGTACTTCTAG |
|  |  | R-ACTACGTCTACAAAATGTCAGTATCAT |
| qPCR | Cutting sites | F-CAGTACTTCTAGCATCAGGTGTTTC |
|  |  | R-AAGGAATGTTGATCCAATAATTACATGGAG |
|  | *Tdg* | F-CTGTGCTACGTCATGCCGTC |
|  |  | R-CGCAAGCTGCAGGTCAAATG |
|  | *Nd2* | F-TCCTGTAATCACAATATCCAGCAC |
|  |  | R-GTTGAGTACGATGGCCAGGA |

**Table S5. PCR primers for Sanger Bisulfite sequencing.**

| **Species** | **Targeting regions** | **Primer sequence (5’-3’)** |
| --- | --- | --- |
| Mouse | Region 2-L | F-AAGGTTTAATTAAATTGTATTTTATATTAT |
|  |  | R-TTCACCTATTTATAAAAATTTAAATTTAAT |
|  | Region 2-H | F-TTATGGAGGTTTAGGTTTAATTGTTAGTGG |
|  |  | R-TCAACTCTTCACACAAACATAACCACTTTA |
|  | lambda | F-GGTTAGAAGTTGTATGTGTTGGAAGT |
|  |  | R-ACTCTAAAAAACACCACCACTAATTA |
|  | *Cox1*-L (sperm) | F-GTTATATAGGAATAGTATGAGTAATAATGT |
|  |  | R-AACAAATCCTACTATAATAACAAACACT |
|  | *Cox1*-H (sperm) | F-ATTGATAGAATATAGTGGAAATGGGTTA |
|  |  | R-CACCCAAAAATTTATATTCTTATCCTCCCA |
|  | *Cox1*-H (*Tet* TKO mESC) | F-GGAAATGTTGAGGGAAGAATGT |
|  |  | R-TATATAAACCCACCACATATTCACAAT |
|  | NUMT | F-TGATAGAATATAGTGGAAATGGGTTATTAT |
|  |  | R-TTCTCCCTACCTTTTCAACATAATAC |
| Human | Region 1-L | F-GTTGAGGTGGATTAAATTAAATTTAGTT |
|  |  | R-CTTAATCTATATTTAACCTAAATTTCTATAAAATT |
|  | Region 1-H | F-TAGTATAAAAGGGGAGATAGGTAGGAGTAG |
|  |  | R-AATTAAAATAAATTAAACCAAACCCAACTA |

**Table S6. Primers for PCR amplification of mtDNA genes.**

| **Targeting regions** | **Primer sequence (5’-3’)** |
| --- | --- |
| *Cox1* | F-GCCGTACTGCTCCTATTATCACTAC |
|  | R-CGTCGTGGTATTCCTGAAAGGC |
| *Cox2* | F-CTAACACATACAAGCACAATAGATGC |
|  | R-TAGTGGAACCATTTCTAGGACAATG |
| *Nd5* | F-GCCCTAACCACATTATTTACAGCT |
|  | R-ATGGTTAGGTTGTTTAGTTCTAGTGC |

**Table S7. qPCR primers for analyzing mtDNA enrichment.**

| **Species** | **Targeting regions** | **Primer sequence (5’-3’)** |
| --- | --- | --- |
| Mouse | *Nd2* | F-TCCTGTAATCACAATATCCAGCAC |
|  |  | R-GTTGAGTACGATGGCCAGGA |
|  | *Tdg* | F-CTGTGCTACGTCATGCCGTC |
|  |  | R-CGCAAGCTGCAGGTCAAATG |
|  | *Gfap* | F-CGTTCCTCCATAAAGGCCCT |
|  |  | R-GAGTGGAGGAGTCATTCGAGAC |
| Human | *ND2* | F-CCCAACCCGTCATCTACTCT |
|  |  | R-GCTTGCGTGAGGAAATACTTG |
|  | *TDG* | F-TTGTGCGGGTGCTTTGAAGT |
|  |  | R-CCATCTCTAGTGGATGTCCGTC |
|  | *GFAP* | F-AGAGGAGACGCATCACCTCC |
|  |  | R-CTTGAAGCCAGCATTGAGTGC |

**Table S8. Published datasets used in this study.**

| **Sample** | **GEO** | **GSM** | **SRR** | **Figure(s)** |
| --- | --- | --- | --- | --- |
| 293T | GSE92310 | GSM2425586 | SRR5100644-48 | S3 |
| 293T |  | GSM2905812 | SRR6426172-74 | S3 |
| 293T |  | GSM2905813 | SRR6426175-77 | S3 |
| 293T |  | GSM2905814 | SRR6426178-80 | S3 |
| A549 | GSE127301 | GSM3633944 | SRR8659902 | 3 |
| mESC | GSE87757 | GSM2339908 | SRR4381931 | 3 |
| PGC M | GSE56697 | GSM1386027 | SRR1286819, SRR1286821 | 1, S1, S2 |
| PGC F |  | GSM1386026 | SRR1286810, SRR1286815 | 1, S1, S2 |
| sperm |  | GSM1386020 | SRR1286766, SRR1286769 | 1, 5, S1, S2 |
| oocyte |  | GSM1386019 | SRR1286759 | 1, S1, S2 |
| 2-cell |  | GSM1386021 | SRR1286781-82 | 1, S1, S2 |
| 4-cell |  | GSM1386022 | SRR1286789, SRR1286791 | 1, S1, S2 |
| ICM |  | GSM1386023 | SRR1286793-94 | 1, S1, S2 |
| E3.5 |  | GSM2577161 | SRR5448119 | 1, S1, S2 |
| E6.5 |  | GSM1386024 | SRR1286802-03 | 1, S1, S2 |
| E7.5 |  | GSM1386025 | SRR1286807-08 | 1, S1, S2 |
| PFC | GSE33722 | GSM830249 | SRR377645 | S1 |
| NA12878 genomic DNA | PRJNA591788 | | SRR10532142 | S5 |
|  |  |  | SRR10532143 | S5 |
|  |  |  | SRR13953196 | S5 |
|  |  |  | SRR10532144 | S5 |
|  |  |  | SRR10532145 | S5 |
